# Transcriptome and Network Analyses of Heterostyly in *Turnera subulata* Provide Mechanistic Insights: Are *S*-Loci a Red-Light for Pistil Elongation?

**DOI:** 10.3390/plants9060713

**Published:** 2020-06-03

**Authors:** Paige M. Henning, Joel S. Shore, Andrew G. McCubbin

**Affiliations:** 1 School of Biological Sciences, Washington State University, PO Box 644236, Pullman, WA 99164-4236, USA; paige.henning@wsu.edu; 2 Department of Biology, York University, 4700 Keele Street, Toronto, ON M3J1P3, Canada; shore@yorku.ca

**Keywords:** *Turnera subulata*, heterostyly, differential gene expression, self-incompatibility, network analysis

## Abstract

Heterostyly employs distinct hermaphroditic floral morphs to enforce outbreeding. Morphs differ structurally in stigma/anther positioning, promoting cross-pollination, and physiologically blocking self-fertilization. Heterostyly is controlled by a self-incompatibility *(S)*-locus of a small number of linked *S*-genes specific to short-styled morph genomes. *Turnera* possesses three *S*-genes, namely *TsBAHD* (controlling pistil characters), *TsYUC6,* and *TsSPH1* (controlling stamen characters). Here, we compare pistil and stamen transcriptomes of floral morphs of *T. subulata* to investigate hypothesized *S*-gene function(s) and whether hormonal differences might contribute to physiological incompatibility. We then use network analyses to identify genetic networks underpinning heterostyly. We found a depletion of brassinosteroid-regulated genes in short styled (S)-morph pistils, consistent with hypothesized brassinosteroid-inactivating activity of *TsBAHD*. In S-morph anthers, auxin-regulated genes were enriched, consistent with hypothesized auxin biosynthesis activity of *TsYUC6*. Evidence was found for auxin elevation and brassinosteroid reduction in both pistils and stamens of S- relative to long styled (L)-morph flowers, consistent with reciprocal hormonal differences contributing to physiological incompatibility. Additional hormone pathways were also affected, however, suggesting *S*-gene activities intersect with a signaling hub. Interestingly, distinct *S*-genes controlling pistil length, from three species with independently evolved heterostyly, potentially intersect with phytochrome interacting factor (*PIF*) network hubs which mediate red/far-red light signaling. We propose that modification of the activities of *PIF* hubs by the *S*-locus could be a common theme in the evolution of heterostyly.

## 1. Introduction

Plants possessing heterostyly exhibit mating types that differ in floral morphology and biochemical mating specificity [1,2]. Species may possess two (distyly) or three (tristyly) floral morphs that exhibit reciprocal positioning of male (anthers) and female (stigma) organs [3]. These traits combine to promote outcrossing and prevent inbreeding [1,4,5]. Often, morphs also exhibit dimorphisms in other reproductive characters, including pollen size/number, stigma size/shape, stigmatic papillae length, and site of incompatible pollen tube inhibition [5,6,7,8,9].

Heterostyly has received considerable attention in several areas of genetics and evolutionary biology. These include supergene inheritance [10,11,12,13], frequency-dependent selection [14], and the evolution and fitness consequences of self-fertilization [15,16,17]. Heterostyly occurs in at least 199 genera in 28 flowering plant families with surprising phenotypic consistency, and has been proposed to have been a major contributor to Angiosperm diversity [18,19,20]. Consequently, these breeding systems present remarkable opportunities to study convergent evolution.

Although heterostyly involves multiple traits it is controlled by a single *S* (self-incompatibility)-locus. Historically heterostyly *S*-loci were viewed as diallelic, short styled (S-) morphs, commonly being heterozygous (*Ss*) and long styled (L-) morphs homozygous recessive (*ss*), but this dogma was recently overturned. *S*-loci of *Primula* [21,22,23,24], *Linum* [25,26], *Fagopyrum* [27,28], and *Turnera* [29], have been at least partially characterized. The studies revealed these *S*-loci to be hemizygous, consisting of a small number of linked genes in S-morph genomes that lack allelic counterparts in L-morph plants.

The nature of *S*-locus- (*S*-) genes identified to date suggests that incompatibility in species with heterostyly is not a “lock and key” recognition system as found in homomorphic SI systems (which do not have morphological variation between mating types) [21,23,30]. In heterostylous species, incompatibility most likely results from physiological differences more analogous to incongruity barriers that reproductively isolate species [31]. These differences result from the action of a small number of *S*-genes, all which have been identified in *Primula* and *Turnera* [21,22,28], providing opportunities to study how these reproductive barriers evolve and operate.

The ~230 kb *S*-locus in *Turnera* contains just three genes [29]. Only one, *TsBAHD,* is expressed in pistils and necessarily determines pistil length and physiology. *TsBAHD* possesses the functional and conserved domains of BAHD acyltransferases and has highest homology to members of this gene family that inactivate brassinosteroids by acylation [23,32]. As a result, *TsBAHD* has been hypothesized to possess brassinosteroid inactivating activity, but as homology-based prediction of BAHD acyltransferase function is unreliable, this needs to be confirmed [33]. Intriguingly, the *Primula CYP734A50 S*-gene was recently demonstrated to control style length by inactivating brassinosteroids [21]. Hence, the independently evolved heterostyly systems of *Turnera* and *Primula* may use a common biochemical mechanism to regulate style length despite recruitment of distinct genes to their *S*-loci [29].

The other two *Turnera S*-genes are expressed in stamens, *TsYUC6* in anthers and *TsSPH1* in anthers and filaments. *TsYUC6* exhibits high homology to YUCCA flavin-dependent monooxygenases, which catalyze the second step in the two-step process of auxin biosynthesis from L-tryptophan [34]. *TsSPH1* is a member of the large plant-specific S-protein homolog (SPH) family named after the *Papaver rhoeas* pistil (homomorphic) SI determinant *PrsS* [35,36,37]. *PrsS* encodes a small secreted polypeptide ligand that interacts with a receptor to activate a signal transduction cascade, leading to programmed cell death in incompatible pollen [38,39]. The function(s) of other members of the SPH family are unknown [36]. The precise roles of *TsYUC6* and *TsSPH1* in determining stamen characteristics are uncertain. Filament length is likely determined by *TsSPH1* as it is missing in L-morph deletion mutants with short filaments, but present in a long-homostyle mutant [29]. *TsYUC6* could potentially regulate pollen size and physiology through auxin pathways, but whether *TsSPH1* may also be involved in these traits is unclear.

Studies in *Primula* [40], *Linum* [25], *Turnera* [41,42] and *Fagopyrum* [43], have identified genes outside the *S*-locus that are differentially expressed (DE) between floral morphs, suggesting that *S*-loci cause differential gene expression. More recent reports have employed transcriptome level expression analyses to study heterostyly in several species including *Lithospermum multiflorum* [44], *Primula vulgaris* [24], *P. maximowiczii* [45], and *P. oreodoxa* [46], but the pathways behind heterostyly remain largely uncharacterized.

Here, we combine the analysis of organ specific transcriptomes with network analyses of differentially expressed genes (DEGs) in an attempt to gain insights into the mechanisms of action of *S*-genes in *Turnera* and the pathways through which they operate. We compare transcript abundance between pistils and stamens of the S- and L-morphs of *T. subulata* at two developmental time points. Then, using the DEG datasets, we investigate whether differential gene expression provides evidence to substantiate hypothesized activities of the recently identified *S*-genes, *TsBAHD*, and *TsYUC6* [29]. Specifically, we consider whether *TsBAHD* controls pistil characters by degrading brassinosteroids and *TsYUC6* stamen characters by synthesizing auxin. This was achieved by assessing whether brassinosteroid and/or auxin related or regulated genes exhibit differential expression in pistils and stamens between the floral morphs. In addition, we assess whether transcriptome data supports the hypothesis that incompatibility in *Turnera* might result from physiological mismatch between pistils with reduced brassinosteroid levels and pollen from anthers with elevated auxin (S-morph), and elevated brassinosteroid pistils and pollen from reduced auxin anthers (L-morph). Lastly, we use the DEG datasets to predict gene networks and pathways underpinning in heterostyly in *Turnera*, the results of which leads us to propose a novel hypothesis regarding the evolution of these breeding systems.

## 2. Results

### 2.1. Differentially Expressed Genes: Identification and Quantification

**Figure 1:**
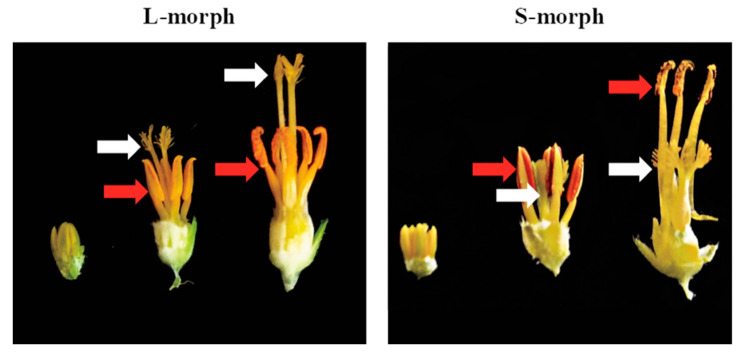
Development of the floral morphs in *Turnera subulata*. The L-morph (left) and S-morph (right) of *T. subulata*. Three developmental time points are depicted from left to right young (3 mm bud), mature (16 mm bud), and open flower. Red arrows highlight the position of the stamens while white arrows the position of the pistil.

Autotetraploid *T. subulata* was chosen as the main focus for this study for a number of reasons. These include, profusion and size of flowers (aiding collection and staging of floral tissues), availability of germplasm (including *S*-locus mutants), and the volume of previous work involving this species [23,41,42,47,48,49,50,51,52]. For *T. subulata*, RNAseq was performed on stamens and pistils (separately) from 3 mm and 16 mm floral buds for each morph. These bud stages (shown in Figure 1) were chosen to represent stages early (targeting morphology) and late in development (targeting mating specificity). RNAseq was also performed on whole buds (minus sepals) of the two morphs of a second, closely related heterostylous species, *T. joelii* (2n) [53,54], allowing us to identify DEGs common to both species (see below).

For each sample ~20 million filtered clean RNA-seq reads were generated (Table S1). Cleaned sequence reads from libraries of both species were mapped to a predicted *T. subulata* transcriptome (enabling direct cross referencing of differentially expressed transcripts between the two species), with mapping rates of 71% for *T. subulata* and 52% for *T. joelii*. Read count tables and differential analysis files were then generated using Blast2GO-Pro (v5.2.5) (Figure S1). Gene expression was compared pairwise between morphs for each developmental stage and organ, and genes statistically differing in expression (adjusted *p*-value < 0.05 and |log_2_FC| > 1.0) retained for further analysis (listed in File S1 Table S2).

**Figure 2:**
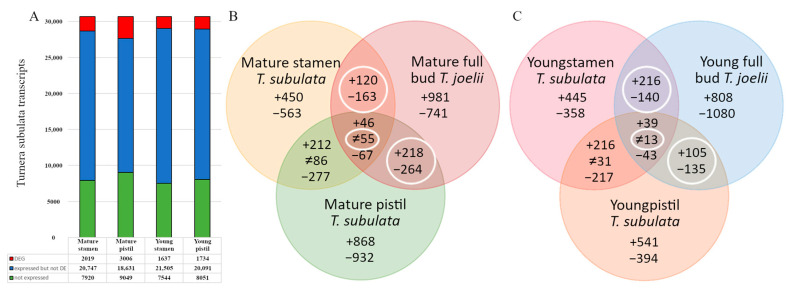
Isolation of organ specific differentially expressed genes (SvL). (**A**) Number of genes identified in each dataset relative to the whole transcriptome, and the fraction designated as DEG between the S- and L-morphs. (**B**,**C**), Venn diagrams depicting comparison of DEGs identified in both *T. subulata* and *T. joelii* at the different developmental stages. Numbers indicate the number of DEGs identified within each dataset (numbers in overlapping sectors indicate DEGs found in all samples overlapping that region). White circles indicate genes retained in the final DEG dataset. Mathematical symbols refer to the expression pattern (SvL) of a given gene. + represents upregulated genes, − represents downregulated, and ≠ genes with reciprocal expression, i.e., upregulated in one tissue type and downregulated in the other.

**Figure 3:**
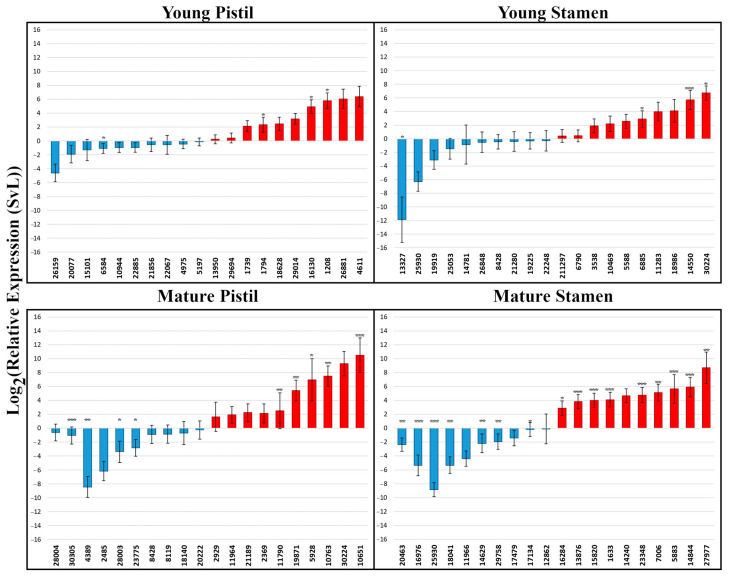
RT-qPCR analysis of select DEGs. Genes identified as up-regulated in the S-morph have been colored red, those down-regulated in the S-morph colored blue. A maximum allowed Cycle Threshold (CT) of 36 was used, samples with higher CT values were assigned values of 36 for graphing purposes. This graph depicts the Log_2_ of relative expression (SvL); also known as the ΔΔCT value. Error bars represent standard error of ΔΔCT values. *p*-values were adjusted using the Benjamini-Hochberg method for FDR correction. * adjusted *p*-value < 0.05, ** adjusted *p*-value < 0.01, *** adjusted *p*-value < 0.001. Descriptions of these genes can be found in File S1.

Initially, 5744 genes were designated as differentially expressed between stamens and/or pistils of the two morphs of *T. subulata*. Despite triplicate biological replicates and stringent criteria to identify DEGs, further investigation (by RT-PCR) suggested this initial dataset contained many false positives (data not shown). Suspecting that allelic variation was a significant factor in false DEG designation (heterostylous species are obligate outbreeders), we added additional screens. *T. subulata* DEGs were cross-referenced with those identified from a second heterostylous species, *T. joelii* (2n) (Figure 2A–C), and genes not designated as DE in both species removed. This resulted in removal of 73.95% (4053) of the DEGs from the initial dataset. *T. subulata* and *T. joelii* are very closely related [53,54] and their *S* locus genes exhibit 94%–96% identity, but mapping *T. joelii* RNAseq reads to the *T. subulata* transcriptome could potentially lead to loss of genes that are polymorphic between the species. To assess whether this was the significance of this issue, all genes removed were assessed for read counts mapped from *T. joelii*. It was determined that only 303 (5.27%) of the genes removed lacked sufficient *T. joelii* reads to enable differential expression analysis (and hence may have been excluded as a result of mapping failure). A peripheral benefit of cross-referencing with *T. joelii* was to remove *T. subulata* DEGs that might have been associated with autoploidy rather than heterostyly in the latter species. As heterostyly in *Turnera* is controlled by three genes with distinct biological activities, DEGs identified as either up- or down-regulated in both stamens and pistils at the same developmental stage were also viewed as likely to have been present for reasons other than heterostyly and removed (195 DEGs). Combined, these screens reduced the total number of DEGs to 1429. To assess the quality of the final datasets, the expression of the top ten up- and down-regulated genes from each dataset was assessed in the relevant organ of each morph by RT-qPCR. In total, 80 DEGs were confirmed to be DE as indicated in the RNAseq datasets (up or down) (Figure 3) providing confidence in these final datasets. Organ datasets were then split into up- and down-regulated sets, for a total of eight, allowing the assessment of processes predicted to be up- or down-regulated in the S-morph.

### 2.2. Identification of Previously Reported Turnera DEGs

As expected, *S*-genes *TsSPH1*, *TsYUC6*, and *TsBAHD*, were all identified in their respective datasets. Three additional genes have been previously reported to exhibit morph specific expression: a style specific polygalacturonase (*TsPG*), a pollen-specific polygalacturonase (*TsPP*) [41], and a style specific α-dioxygenase (*TsαDOX*) [42], so we searched for these genes in our data.

Two neighboring (on a genome scaffold) predicted genes were identified with high identity to *TsPG*, Tsub_00011274-RA aligns to the front of *TsPG*, and Tsub_00011275-RA to the end, suggesting both contain ORFs of *TsPG* (supported by their similar Log_2_FCs in all analyses). In our datasets *TsPG* was up-regulated in young and mature pistils and down-regulated in mature stamens of the S-morph. In young stamens transcript counts were <10 in all replicates, consistent with no expression in this sample. A full-length predicted gene for *TsPP* was identified (Tsub_00009438-RA) and determined to be expressed only in mature S-morph stamens, consistent with the previous report [41]. *TsαDOX* was also identified (Tsub_00010763-RA) and determined to be up-regulated in S-morph mature pistils, this transcript is present in other samples, but at dramatically lower read counts (<120 v’s > 70,000), consistent with the previous report that *TsαDOX* expression is specific to S-morph styles [42].

### 2.3. Analysis of Differential Expression

Of the 1429 genes identified as DE in at least one comparison, 369 were DE in young stamens, 338 in mature stamens, 253 in young pistils, and 538 in mature pistils (Figure 2B). Combining developmental stages, 685 genes were DE in stamens and 760 in pistils. Of these, 1311 were present in more than one binary comparison, and 121 exhibited reciprocal differential expression between pistil and stamen tissues (Figure 2B,C). One aim of this study was to identify gene networks involved in heterostyly in *Turnera* to enable development of testable hypotheses. Tools that facilitate such analyses are essentially non-existent for non-model species. One approach to resolve this problem is to convert non-model species datasets into *Arabidopsis* genes. A caveat to this approach is the inference of orthology between *Arabidopsis* and *Turnera* homologs. Though BLAST scores are not robust metrics for predicting orthology, it is reasonable to assume designations to be accurate in regard to gene family, if not specific isoform. Consequently, though caution is needed in extrapolating results in regard to specific isoform, for predicting gene function and enabling hypothesis development these designations have considerable value. We performed this conversion by identifying the *Arabidopsis* homolog with the lowest e-value (using BLASTp) for each DEG. We initially performed ClueGo enrichment to assess enriched and depleted Gene Ontology (GO) terms in the datasets.

### 2.4. ClueGO Analysis of Enriched and Depleted GO Terms

All converted datasets were analyzed using the Cytoscape application coupled with the ClueGO plug-in, to identify enriched GO terms and clusters. A comprehensive list of enriched GO terms and clusters can be found in File S2. GO terms and genes in up-regulated datasets are referred to as enriched, and those in down-regulated datasets as depleted.

**Figure 4:**
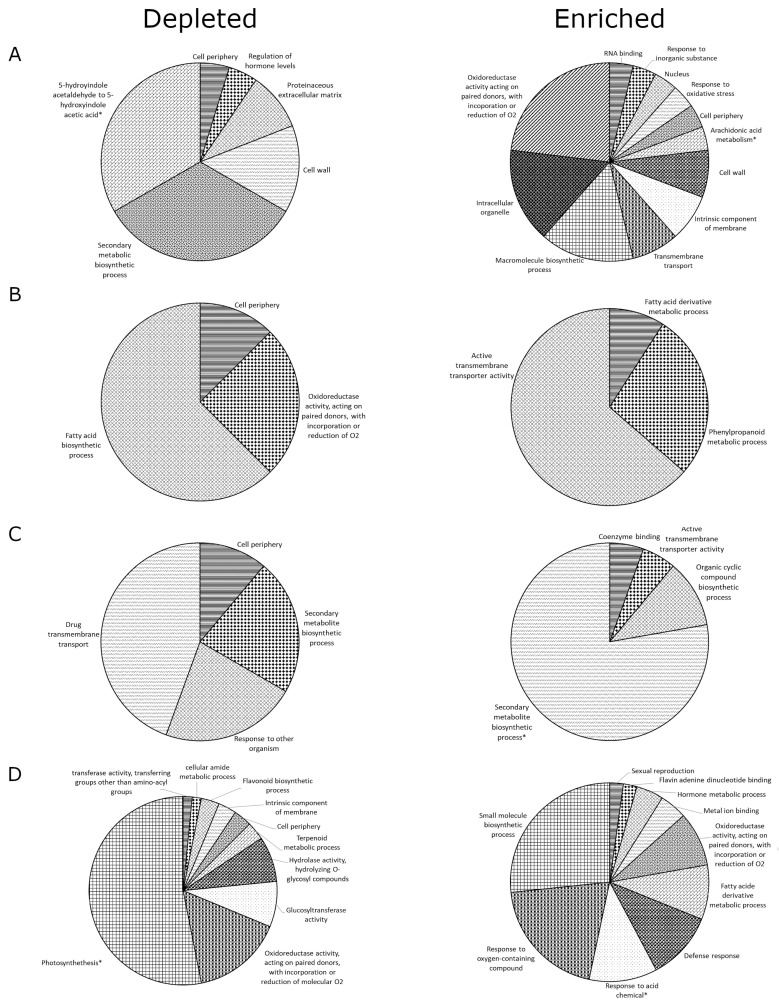
Enriched and Depleted ClueGO clusters of up-regulated DEGs (SvL). Pie charts depicting ClueGO generated clusters and overall percentage of GO terms associated with each cluster. Clusters contain GO terms from young stamen (**A**), mature stamen (**B**), young pistil (**C**), and mature pistil (**D**) that have commonality. Enriched and depleted are relative to S- vs L-morph expression levels; i.e., enriched terms are upregulated and depleted terms downregulated in the S-morph. * represents clusters containing KEGG or Reactome pathways. (Individual GO terms have been represented using bar graphs generated via ClueGO, provided in Figure S2. For a comprehensive list of the clustered GO terms, see File S2.) All clusters had a *p*-value ≤ 7.28 × 10^−4^.

In young S-morph stamens, 26 GO terms were enriched, clustering into twelve groups (Figure 4A). Fifty-eight DEGs associated with the term cell periphery (GO:0071944) and 16 with transmembrane transport (GO:0055085). *TsYUC6* associated with seven terms in this dataset (Table S2). Twenty-one GO terms were depleted, clustering into six groups (Figure 4A). One depleted cluster was composed entirely of pathways related to the conversion of 5-hydroxyindole acetaldehyde to 5-hydroxyindole acetic acid. Again, many genes associated with the term cell periphery were depleted. The term regulation of hormone levels (GO:0010817) was also depleted.

In mature S-morph stamens, eleven GO terms were enriched, clustering into three groups (Figure 4B). Enriched clusters included fatty acid derivative metabolic processes (GO:1901568) and active transmembrane transporter activity (GO:0022804), which encompassed the majority of enriched GO terms and included several ABC transporters. Eight GO terms were depleted in mature stamens, clustering into three groups (Figure 4B). Forty-five genes associated with the cell periphery (GO:0071944), and again several ABC transporters associated with this term.

In young S-morph pistils, eighteen GO terms were enriched, clustering into four groups (Figure 4C). Moreover, 77% of enriched terms clustered under the term secondary metabolites. Several of these related to flavonoids, including anthocyanidin (GO:0046283), phenylpropanoid metabolic (GO:0009698), and biosynthetic processes (GO:0009699). Nine terms were depleted and clustered into four groups (Figure 4C). The largest cluster, drug transport (GO:0015893), included several ABC transporters. *TsBAHD* did not associate with any enriched term in either dataset.

In mature S-morph pistils, 40 GO terms were enriched, clustering into 10 groups (Figure 4D). The term hormone metabolic processes (GO:0042445), including jasmonic acid signaling (GO:0009867), fatty acid derivative biosynthesis (GO:1901570), and metabolic (GO:1901568) processes were enriched. A total of 64 GO terms were depleted, clustering into ten groups (Figure 4D). 53% of depleted GO terms are related to photosynthesis. Both flavonoid metabolic process (GO:0009812) and biosynthetic process (GO:0009813) terms were depleted.

### 2.5. Kyoto Encyclopedia of Genes and Genomes (KEGG) Analysis

A total of 675 KEGG Orthology (KO annotations were assigned to the 8 datasets (Figure S4a). At the global level, overview maps were similar between samples, but young tissue types had an overview map specific to their enriched and depleted datasets. For young pistils this was degradation of aromatic compounds (01220), and for young stamen 2-oxo-carboxylic acid metabolism (01210) (Figure S4b). The terms metabolic pathways and biosynthesis of secondary metabolites were present in all eight DEG datasets (Figure S4b; File S3). Genes involved in BR biosynthesis (map:00905) were identified in depleted datasets of both young and mature pistils and the enriched set of mature pistils consistent with *TsBAHD* being involved in BR metabolism (File S3). Eleven DEGs with BR related GO terms were identified in pistils, four enriched and seven depleted. *DWF4/Cyp90B1*, *FER*, *At4g30410,* and *MYB56* were enriched. *DWF4* is negatively regulated by BR and its upregulation consistent with reduced BR content in S-morph pistils. *At4g30410* and *MYB56* are both antagonists of BR signaling, and their upregulation is consistent with reduced BR signaling. *FER* is required for BR stimulated elongation of hypocotyls [55]. Amongst the depleted DEGs, several are induced by BRs (*CYP716A1*, *CYC3; 1,* and *EXO*), one a transcriptional repressor of auxin and BR regulated genes (*PAR1*) and two encode BR degrading enzymes (*BAS1* and *BEN1*), again consistent with a reduced BR environment in S-morph pistils. Overall, these results support the hypothesis that *TsBAHD* functions as a BR degrading enzyme. As a member of the *YUCCA* gene family, *TsYUC6* is hypothesized to catalyze conversion of indole-3-pyruvate (IPA) to indole-3-acetic acid (IAA) [56]. Consistent with this hypothesis, genes in tryptophan metabolism (map:00380), which includes this conversion, were identified in both young and mature stamen enriched DEGs (File S3). Other genes involved in tryptophan metabolism also occur in the depleted dataset of young stamens. These include *YUC2*, which acts redundantly with *YUC6* in *Arabidopsis* [57], and components of alternative pathways to auxin synthesis, consistent with negative feedback down-regulation resulting from auxin production by *TsYUC6.*

In stamens, 16 DEGs (in addition to *TsYUC6*) were annotated with auxin related GO terms (eight enriched, eight depleted). Of the enriched genes, four are auxin induced (*AIR3*, *IAA7, SAUR27*, *DFL1*) and four involved in auxin transport (*PIN6*, *TT7*, *TT4*, *ABCB11*), consistent with a higher auxin environment in S-morph stamens. For depleted DEGS, the situation was less distinct. One, SGR5, is involved in regulation of auxin gradients regulating *YUC* gene mediated auxin synthesis and its down-regulation consistent with negative feedback in a high auxin environment. Two (*BRU6* and *IAA14*) are auxin upregulated, but were depleted, as both are negative regulators of auxin signaling at low auxin levels, depletion might reflect abnormally high auxin levels in S-morph relative to L-morph anthers. Another two (*LAX3* and *ABCG37*) are auxin transporters. Of the others, *AGO1* a key player in microRNA pathways, regulates auxin-induced adventitious root formation, and *ASK2* is a component of an SCF complex required for auxin mediated degradation of repressors of the auxin response.

We next sought evidence for reciprocal changes in BRs and/or auxin level between pistils and stamens of the two morphs, which might potentially contribute to physiological incompatibility. For this analysis we added back DEGs that were enriched or depleted in both stamens and pistils of the S-morph. In stamens, four BR related DEGs were identified, *At5g36130* was enriched, and *CDL1*, *PAR1,* and *EXO* depleted (the latter two being also depleted in pistils). This suggests that BR levels were reduced in S-morph stamens as well as pistils. In pistils, 16 auxin-related DEGs were identified, only one common to stamens (*IAA14*, depleted in both tissues). Enriched DEGs included auxin biosynthesis genes (*AAO2*), auxin induced genes (*At5g54490*, *WRKY23*, *DFL1*), genes involved in auxin signaling (*ABCB19*, *AFB3*, *MYBR1*, *At3g22810*), and an auxin influx carrier (*LAX2*). Depleted DEGs included several auxin transporters (*NRT1.1*, *CYP711A1*, *TT4*, *TT7*, *At5g14090*), and a transcriptional activator of *YUCCA* genes (*SRS7*). Overall, these results suggest that S-morph pistils have elevated auxin levels relative to L-morph pistils, but that this is achieved via an alternative auxin synthesis pathway (involving *AAO2* rather than *YUCCAs*) and possibly decreased auxin transport.

Given the small number of genes controlling heterostyly, an interesting finding was that a substantial number of DEGs (22) were annotated with GO terms related to other plant hormone pathways. This suggests that the activities of *TsBAHD*, and *TsYUC6,* and *TsSPH1* in pistils and stamens respectively, have transcriptional repercussions beyond direct BR and auxin regulation. The diversity of plant hormone pathways represented in the DEGs suggest that activities of the *S*-genes intersect with major developmental networks or signaling hub(s). With this in mind, we investigated potential gene networks represented within the datasets.

### 2.6. Prediction of DEG Association Networks in Stamens and Pistils

STRING analysis utilizes known and predicted genetic association data to identify genetic networks in novel datasets. The analysis predicts both direct (physical) interactions and indirect (functional) interactions, using data including co-expression, detection of shared selective signals across genomes, text-mining of scientific literature and computational transfer of known interactions between organisms based on gene ontology [58]. Young and mature DEG datasets were combined, including enriched and depleted DEGs for each tissue, and used to generate organ specific gene association networks using STRING. As differential expression was the sole criterium used to identify the genes included, these analyses were expected to identify components of pathways involved in heterostyly, but not necessarily entire pathways. The networks generated can be found in Figures S5 and S6 and a comprehensive list of predicted associations in File S4.

STRING analysis identified 6740 interactions between 606 designated *Arabidopsis* homologs in pistils and 3964 interactions between 534 designated *Arabidopsis* homologs in stamens, and overall networks for each tissue are available in Figures S5 and S6, File S4, and the NDEx repository (Table S3). NCMine software extracts subgraphs from networks based on node-weighting using degree centrality, reporting subgroups as functional modules [59]. Given the number of interactions predicted, we focus on functional clusters directly involving *S*-genes identified. Descriptions of gene function are derived from annotations in STRING unless otherwise referenced.

#### 2.6.1. Predicted TsBAHD Gene Network Associations in Pistils

Of the 606 DEGs in pistils, 25 are first neighbors with *TsBAHD* (Figure S7), meaning they directly form an edge with *TsBAHD* connecting it with the rest of the network. Four of these have connections to BR signaling, as *DWF4* (enriched), *AHL24* (enriched), *EIR1* (depleted) are BR induced auxin carriers, and *At3g21710* (*VUP1*) a BR/auxin regulator (enriched). *DWF4* encodes a C-22 hydrolase critical to BR synthesis, is negatively regulated by BR and has been suggested to be a point of cross-talk between BR and auxin pathways [60]. *AHL24* is a member of the AT-hook-containing protein family, which inhibit transcription of genes involved in auxin synthesis and auxin-mediated cell elongation in hypocotyls [61,62,63,64]. Several enriched DEGs genes annotated as being involved in abscisic acid (ABA) signaling (*DOX1*, *PHO1* and *RBHOH*) were also in this group.

Eight clusters containing *TsBAHD* were identified, containing a combined total of 52 DEGs. All are available on the NDEx repository, in the interest of brevity, we focus on the two subnetworks (Figures S8 and S9) with highest clustering coefficients and lowest heterogeneity scores, and DEGs of particular note.

**Figure 5:**
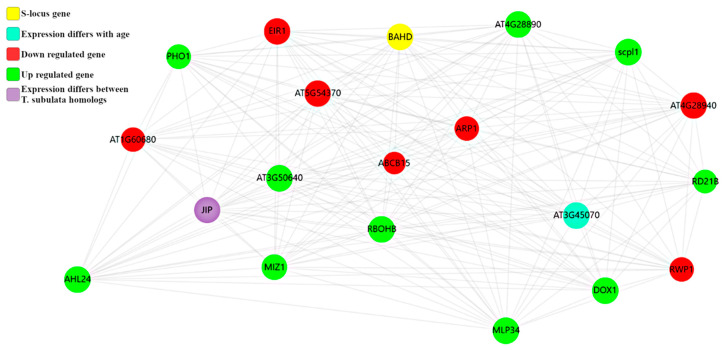
Representative String Network: Predicted *TsBAHD* gene network association cluster 4. Cluster 4 is of particular interest as it contains several hormone related genes and a member of the AHL family, which we hypothesize may be important for female morphological characteristics.

Cluster 4 (clustering coefficient 0.962, heterogeneity 0.066) contains several DEGs of interest (Figure 5). *AHL24* (enriched) is involved in regulation of hypocotyl elongation as noted above. *MIZ1* (enriched) negatively regulates cytokinin sensitivity in root development. *DOX1* (enriched) promotes establishment of systemic acquired resistance, in a salicylic acid-dependent manner, in response to incompatible interaction. This gene is also implicated in the negative regulation of ABA-mediated signaling pathway. *EIR1* (depleted) is an auxin efflux carrier 

Cluster 8 (clustering coefficient 1.0, heterogeneity 0.0) contains only two DEGs in addition to *TsBAHD*, *FAF3* which is involved in repressing the WUS homeobox gene controlling the stem cell pool, and *ABCB15* a proton ATPase that can transport auxin [65].

#### 2.6.2. Predicted TsYUC6 Gene Network Associations in Stamens

Of the 534 DEGs that form networks in stamens, 22 are predicted to be first neighbors with *TsYUC6* (Figure S10). Six of these are annotated as having functions in auxin signaling, as *PIN6* (enriched) is an auxin efflux carrier, *SAUR27* (enriched) an auxin responsive gene, *BRU6* (depleted) catalyzes synthesis of indole-3-acetic acid (IAA)-amino acid conjugates (a mechanism for plants to inactivate excess auxin), *Cyp79B2* (depleted) converts tryptophan to indole-3-acetaldoxime a pre-cursor for IAA in an alternative auxin synthesis pathway, *SGR5* (depleted) is an auxin gradient regulator, and *LAX3* (depleted) an auxin transporter. Several other first neighbors of *YUC6* are involved in other plant hormone pathways: *GA20OX1* (enriched) in gibberellic acid (GA) synthesis, *IPT5* (enriched) in cytokinin synthesis, and *CDL1* (depleted) in BR synthesis.

Thirteen clusters were identified amongst the 77 DEGs predicted to participate in sub-networks (Figures S11–S14) containing *YUC6* (NDEx repository). Cluster 4 (clustering coefficient 0.8, heterogeneity 0.471) contains several genes involved in regulating GA homeostasis, *GA2OX1* (enriched) is involved in GA synthesis, and *GA2OX4* (depleted) and *GA2OX6* (enriched) have similar activities in interconverting GA variants.

Cluster 5 (clustering coefficient 0.8, heterogeneity 0.328), contains two genes involved in cytokinin homeostasis, *IPT5* (enriched) is involved in cytokinin synthesis and *LOG5* (enriched) is a cytokinin activating enzyme. In addition, this cluster contains a down-regulated auxin transporter-like protein, *LAX3*.

Cluster 6 (clustering coefficient 0.833, heterogeneity 0.2) contains two genes involved in auxin synthesis, both depleted (*BRU6* and *YUC2*), presumably a result of negative feedback resulting from auxin synthesis by *TsYUC6*.

Cluster 13 (clustering coefficient 0.423, heterogeneity 0.413) contains several genes involved with auxin. *ABCG37* (depleted) a negative regulator of auxin polar transport inhibitors, *MIZ1* (depleted) which plays a role in lateral root development by maintaining auxin levels as well as negatively regulating cytokinin sensitivity in root development. Lastly, *RCI3* (enriched) which is involved in auxin catabolism.

#### 2.6.3. Predicted TsSPH1 Gene Network Associations in Stamens

Only four genes are predicted to be first neighbors with *TsSPH1* (Figure S15). *TsSPH1* is hypothesized to regulate filament elongation [29]. Three formed a single cluster (clustering coefficient 0.833, heterogeneity 0.2) (available on the NDEx repository; Figure S16). These were *At1g59835* (*CEP2*) (enriched) and two *RAPID ALKALINIZATION FACTOR-LIKE (RALFL*) proteins, *RALFL7* (depleted) and *RALFL16* (enriched). All three encode small extracellular signaling polypeptides.

*CEP2* encodes a small extracellular peptide ligand mobile and is expressed in *A. thaliana* filaments. Some CEPs have been shown to bind to leucine rich repeat receptor kinases to negatively regulate root growth [66]. Both *RALFL7* and *RALFL16* are type IV RALFs, a clade predominantly expressed in *Arabidopsis* inflorescence tissues, and *RALFL16* is exclusive to floral tissues [67]. FERONIA is the only known RALF receptor, and binds RALF to activate a signaling cascade that inhibits cell elongation [67,68]. Though *RALFL7* is depleted and *RALFL16* enriched in S-morph stamen, read counts suggest that overall RALFL proteins are reduced by ~10 fold in S-morph stamens (long filaments), relative to L-morph (short filaments). This leads us to speculate that depletion of *RALFL7* could be integral to promoting cell elongation in S-morph filaments by removing inhibition of FERONIA.

#### 2.6.4. Genes and Networks with Reciprocal Differential Expression between Stamens and Pistils

We hypothesized that SI in *Turnera* might result from reciprocal differences in gene expression in stamens and pistils. Consequently, we investigated potential gene networks enriched in one organ and depleted in the other. To identify such networks, we compared stamen and pistil datasets using DyNet. A total of 385 interactions between 128 DEGs are shared between the organs. Of these, 76 showed reciprocal expression and 132 predicted interactions were identified. These genes clustered into 5 GOGroups (Table S4). The DEGs clustered under the terms catalytic activity, nucleus, cell wall organization, terpenoid biosynthetic process, and intrinsic components of membranes. These results suggest that there may be reciprocal differences in isoprenoid and terpenoid metabolism between stamens and pistils of the two morphs. The majority of DEGs listed as intrinsic membrane components are membrane transporters, a number of which possess ATPase activity. These could potentially be related to reduced cell size in pistils and increased pollen/filament cell size in S- relative to L-morph flowers. The presence of several ABC transporters in this group is also intriguing as these could potentially be involved in the transport of auxin or peptide ligands.

## 3. Discussion

Several insights into the mechanisms underpinning heterostyly in *Turnera* were gained in this study. Empirical evidence, in the form of differential gene expression, was found for the hypothesized enzyme activities of the *S*-genes *TsBAHD* and *TsYUC6*. Specifically, the identity and changes in expression of DEGs in pistils support lower brassinosteroid levels in S- relative to L-morph pistils, and higher auxin levels in S- relative to L-morph anthers. Somewhat surprisingly, the data also suggest that auxin and brassinosteroid levels are altered in a consistent manner in anthers and pistils, leading us to extend our hypothesis that incompatibility in *Turnera* results from physiological hormone mismatch between pollen and pistils. Lastly, evidence was found for other hormone pathways being perturbed by the action of the three *S*-genes, suggesting that a network signaling hub might underpin heterostyly. The identities of DEGs forming genetic functional clusters directly involving *S*-genes were determined by network analysis, generating results that lead us to propose a novel hypothesis concerning the mechanism and evolution of heterostyly below.

Robust datasets are critical for meaningful bioinformatic analysis, and false positives were a significant issue in our initial DEG datasets. This was most likely due to the inherent heterozygosity of obligately outbreeding species causing miss-mapping to the transcriptome. This being the case, increasing the number of biological replicates was unlikely to solve this issue. Our approach of cross-referencing DEGs between two heterostylous species (which differ in the genes affected by issues caused by heterozygosity) dramatically improved the quality of the DEG analysis, as confirmed by assaying eighty DEGs by qPCR. The resulting datasets were inclusive in that they included the three *S*-genes and all previously identified DEGs, and differential expression and tissue specificity of these genes was consistent with previous reports with a single exception. *TsPG* a polygalacturonase previously reported to be style specific [41], was found in this study to be expressed in mature stamen as well as pistils. As the previous report assessed expression in pollen rather than stamens, this discrepancy is likely because *TsPG* is expressed in mature anther or filament tissues. This consistency between our results and published data provided confidence that our DEG datasets were robust, and included the majority of differentially expressed genes, and hence further analyses would provide meaningful information.

The analyses provided support for the hypothesis that *TsBAHD* functions as a BR degrading BAHD acyltransferase to control pistil length. In S-morph pistils, genes involved in BR biosynthesis were depleted, genes negatively regulated by BR (*DWF4*) and antagonists of BR signaling (*At4g30410* and *MYB56*) enriched, and a BR regulated gene (*PAR1*) and BR degrading enzymes (*BAS1* and *BEN1*) were depleted. These results are consistent with a reduced BR environment in S-morph, relative to L-morph, pistils. A recent study of pistils of heterostylous *Primula oreodoxa* reported that genes related to plant hormone signal transduction and BR biosynthesis pathways were also differential in *Primula* pistils [46]. As style length in *Primula* is governed by the presence of the BR degrading enzyme CYP734A50 [21], the data from Primula, combined with the results presented herein support that a common mechanism underlies pistil characteristics in *Primula* and *Turnera*.

Support was also found for the hypothesis that *TsYUC6* controls pollen and/or stamen characteristics through auxin mediated pathways [29]. In *Arabidopsis*, *AtYUC6* and *AtYUC2* act redundantly to regulate pollen development, filament elongation, and anther dehiscence [57,69,70]. As a *YUCCA* gene family member, *TsYUC6* likely catalyzes conversion of indole-3-pyruvate to indole-3-acetic acid [56]. We found evidence in the DEG datasets for elevated auxin levels in S-morph stamen. Genes involved in tryptophan metabolism, which includes *YUCCA* mediated auxin synthesis, were identified in both young and mature stamen enriched DEGs. Other genes associated with tryptophan metabolism were depleted in young stamens, including *YUC2* and components of alternative pathways to auxin synthesis, likely a result of negative feedback caused by auxin production by *TsYUC6.* Transcriptional evidence for the presence of elevated auxin in S-morph anthers included enrichment of auxin induced genes (*AIR3*, *IAA7, SAUR27*, *DFL1*), genes involved in auxin transport (*PIN6*, *TT7*, *TT4*, *ABCB11*), and depletion of the auxin repressed gene *SGR5*, auxin transporters (*LAX3* and *ABCG37*), and *Cyp79B2*, which converts tryptophan to indole-3-acetaldoxime. *BRU6* and *IAA14* were also depleted, and both are rapidly but transiently induced at low auxin levels [71] and their depletion likely reflects long term auxin elevation in S-morph anthers. Though it is not immediately clear why some auxin transporters would be enriched and others depleted, overall, these results are consistent with auxin being higher in S- than L-morph anthers, consistent with *TsYUC6* mediating at least some aspects of stamen phenotype by auxin synthesis.

**Figure 6:**
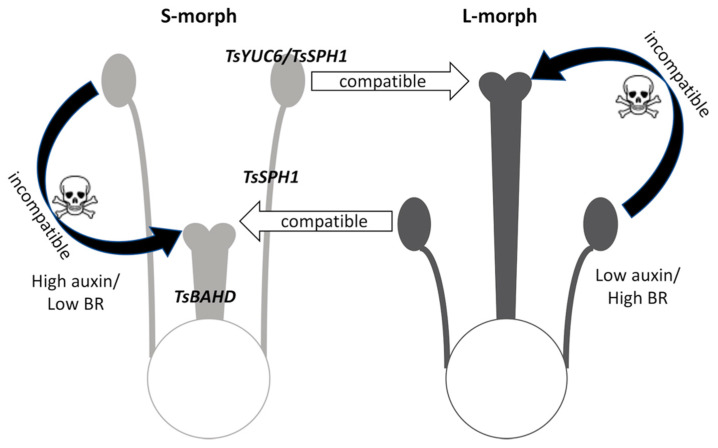
Hypothesis for the basis of self-incompatibility in *Turnera*. Pale grey indicates stamens and pistil with relatively low brassinosteroid and high auxin concentration in the S-morph flowers, dark grey the relatively high brassinosteroid and low auxin concentration in stamens and pistil of the L-morph. It is hypothesized that pollen developing in one hormone environment cannot grow on/in pistils with the same hormone environment (self- or within morph pollinations) and dies after within-morph or self-pollination. In contrast, cross-pollination to pistils with the opposite hormone environment leads to successful pollen tube growth and fertilization. *S*-gene labels indicate the tissues in which they are expressed in the S-morph.

The paucity of functional characterization of *SPH1* gene family members makes hypothesizing a mechanism of action for *TsSPH1* challenging. Network analysis identified genes that might act in genetic networks with *TsSPH1* to influence stamen development. That *RALFL7* is part of a network with *TsSPH1* and was depleted is interesting. RALF activates a signaling cascade mediated by the receptor kinase FERONIA to inhibit cell elongation [67,68]. Whether *RALFL7* possesses this activity is unknown, but reducing RALFL7 could potentially promote cell elongation by releasing inhibition of FERONIA resulting in the long S-morph filament phenotype. We previously proposed that incompatibility in *Turnera* might result from physiological mismatch between low BR pistils/high auxin pollen (S-morph) and high BR pistils/low auxin pollen (L-morph). We identified a number of genes that exhibited reciprocal differential expression between pistils and stamens, as might be expected of genes contributing to a physiological barrier preventing self-fertilization. Genes involved in terpenoid pathways and membrane transporters were found to be highly represented but no pathway with clear implications to pollen-pistil compatibility was identified. Interestingly, however, DEGs were identified that suggested that auxin and BR are altered in a consistent manner in pistil and stamen tissues within a floral morph. despite different *S*-genes being expressed in each tissue. This suggests that BR was reduced and auxin elevated in both pistils and stamens in S-morph relative to L-morph flowers, and that this is caused by a different set of genes in each tissue. This is consistent with, but extends upon, our previous hypothesis that reciprocal hormone environments between morphs might be a responsible for compatibility [29]. The new data suggest that pollen that developed in a low BR/high auxin environment (S-morph) is incompatible with low BR/high auxin S-morph pistils but compatible with high BR/low auxin L-morph pistils, and vice versa (Figure 6).

Beyond supporting the hypothesized enzyme activities of *TsYUC6* and *TsBAHD*, the results of this study provide evidence that auxin levels in stamens and BR levels in pistils are not the only hormonal differences caused by the *S*-locus. Rather, multiple hormone pathways are affected in both organs, suggesting that auxin and BR signaling in stamens and pistils (respectively) intersect with additional signaling networks. To investigate this further, we used transcriptome data to predict gene networks directly involving *S*-genes.

A particularly noteworthy DEG found in these networks was Tsub_00021189 (an *AHL* family member), which was upregulated in S-morph pistils. *AHL*s regulate homeostasis of a variety of plant hormones including GA [72], jasmonic acid [73], cytokinin [74], auxin [63,64], and BR [62,64]. They anchor DNA to the nuclear matrix by binding AT rich conserved scaffold matrix nuclear attachment regions, to affect gene regulation through chromatin remodeling [63]. *AHLs* interact to form homo-/hetero-complexes, which complex with other nuclear proteins that modulate plant growth and development in response to environmental signals [62]. Two members of this gene family, namely SUPPRESSOR OF PHYTOCHROME B-4 #3 (*SOB3*) and ESCAROLA (*ESC*), act redundantly to repress hypocotyl elongation in light grown *Arabidopsis* seedlings [61]. Knocking-out additional *AHL* family members, increases phenotype severity (hypocotyl length) [62]. Intriguingly, seed set is reduced in Arabidopsis plants overexpressing a dominant mutant *SOB3* allele, but whether this is caused by breakdown of embryo development or a pollination defect (potentially analogous to SI in heterostyly) has not been investigated [61]. We propose that Tsub_00021189 may play a key role in the short pistil phenotype.

There has been much recent progress made in elucidating pathways in plant hormone signaling and major signaling hubs identified. Surveying the biological effects of these pathways, the genetic components of signaling hubs relative to the DEGs identified in this study, and the identity of *S*-genes in this and other species, leads us to speculate a common theme in the evolution of heterostyly, at least in regard to pistil characters. We hypothesize this to be recruitment to the *S*-locus of genes that intersect with, and modify the action of, phytochrome interacting factor (PIF) signaling networks.

Phytochrome associated pathways can modify stamen and pistil morphologies, as both can be modified by red/far-red light levels [75]. *Brassica rapa* plants exposed to low red/far-red have shorter pistils and longer stamens than plants exposed to high red/far-red, increasing herkogamy in a manner analogous to heterostyly [75]. Further, PIF signaling hubs intersect with and affect hormone pathways including GA, auxin, BR, ABA, and jasmonic acid [76] in a manner comparable to the differential expression observed in this study. Several gene families represented by DEGs in this analysis are significant players in PIF networks, (including *SAURs*, *IAAs*, *DWFs*, *GA20OXs*, *PINs*, *AHLs*). In particular, *AHLs* are implicated in *PIF* pathways regulating cell elongation in hypocotyls [61,64,77]. Combined, these factors make PIF signaling networks attractive candidates to be hubs involved in heterostyly.

Interestingly, all *S*-genes that control pistil characteristics identified to date potentially intersect with *PIF* hubs. In *Primula* and *Turnera*, pistil length appears to be mediated by BR degradation, albeit by genes encoding different enzymes [21,23], and BR related differential expression in pistils in *Turnera* also occurs in *Primula* [46]. In *Fagopyrum*, the only other heterostylous species for which a pistil *S*-gene has been identified, style length is regulated by *S*-Locus Early Flowering 3 (*S-ELF3*) [78]. *S-ELF3* is a homolog of *Arabidopsis ELF3,* a nuclear protein that regulates flowering by interacting with the PIF4 network [79]. In each case the activities of pistil *S*-genes are analogous to at least some of the actions of red/far-red light on a PIF network. As PIF hubs involve a large number of interacting factors, there are numerous ways, involving many different genes, by which their activities can be modulated. Heterostyly has evolved frequently and seemingly independently with remarkable phenotypic consistency [20], and the large array of genes that participate in PIF networks provide attractive potential targets through which this consistency might evolve. The recent development of transformation systems for heterostylous species [79,80] has provided key tools to enable testing of this hypothesis by direct manipulation of *AHLs* and other components of *PIF* pathways to investigate their potential roles in heterostyly.

## 4. Materials and Methods

### 4.1. Tissue Collection, RNA Isolation, and RNA-Seq

*Turnera subulata* (autotetraploid) and *T. joelii* (2n) were grown in greenhouse conditions. Tissue samples were chosen to represent stages early (targeting morphology) and late (targeting mating specificity) in development. For *T. subulata*, stamens and pistils were collected separately from 3 mm and 16 mm floral buds for each morph. For *T. joelii*, whole 3 mm and 16 mm flower buds (sepals removed) were harvested. RNA was isolated using Concert Plant RNA Reagent following the “Small-Scale Isolation” protocol (Invitrogen, USA). RNA was treated with DNase I (Thermoscientific, Carlsbad, CA, USA) and cleaned using an RNA-Clean & Concentrator-5 kit (Zymoresearch, Irving CA, USA) following the manufactures’ protocol. RNA integrity was measured using an RNA Nano 6000 Assay Kit with an Agilent Bioanalyzer 2100 (Agilent Technologies, Santa Clara, CA, USA). Library construction and sequencing by Illumina PE150 was performed by Novogene (Sacramento, CA, USA). Paired-end reads with a Phred score of <30 were filtered out of the final data set (Table S1). Blast2GO-Pro (v5.2.5) was used to map RNA-seq data to a reference *T. subulata* transcriptome to quantify transcript abundance and generate read count tables.

### 4.2. Transcriptome Annotation

A transcriptome of 30,686 genes, predicted from the genome of *T. subulata* (2n) [Shore et al. in preparation] was annotated using Blast2GO-Pro (v5.2.5) (NCBI Bioproject PRJNA589060, Genbank Accession Number GICK01000000). BLASTx searches were restricted to Viridiplantae, default settings were used for all parameters.

### 4.3. Quantification of Transcript Abundance and Differentially Expressed Gene (DEG) Identification

RNAseq reads were mapped to the transcriptome using Blast2GO-Pro (v5.2.5) [81] using default parameters, to generate read count tables for each sample. These were then used to identify differentially that were expressed between S- and L-morphs using the general linearized model likelihood method coupled with relative log expression normalization also using Blast2GO-Pro (v5.2.5), which employs edgeR for DE analysis [81]. Cut-off parameters for DEGs were an absolute log_2_ fold change of >1 and a Benjamini–Hochberg adjusted *p*-value of < 0.05. MA plots were generated using Blast2GO-Pro (v5.2.5) and heatmaps with InCHlib using the Log_2_(CPM) and Z-score option [82]. *T. subulata* DEGs were cross-referenced to *T. joelii* DEGs and only those common to both retained. Removed genes were assessed for the number of read counts mapped from *T. joelii* to quantify potential interspecies mapping failure. Genes that shared expression patterns between different organs within a developmental stage were considered to possibly have been designated DEGs as a result of allelic variation and removed (but later replaced for KEGG and STRING analyses). Blastx was used to identify closest *Arabidopsis thaliana* homologs of DEGs for downstream analyses (with cut-offs of >35% query coverage and e-value < 0.05).

### 4.4. RT-qPCR Analysis of DEGs

qPCR was performed for the top ten up- and down-regulated genes from each dataset. RNA was purified and DNase I treated as above. cDNA was synthesized using the SensiFAST cDNA synthesis kit and manufacture’s protocol (Bioline, UK). All qPCR reactions used a SensiMix SYBR Low-ROX kit with a slight modification of the manufacturers’ protocol (Bioline, UK). Primers (5 µM each) and 20 ng RNA were combined in 20 µL reactions. Samples were run on an ABI 7500 fast machine (ABI, Vernon, CA, USA) and normalized using UEV1D and β tubulin. Results were analyzed using ExpressionSuite (v1.1) and DataAssist (v3.01) (ThermoFisher, Waltham, MA, USA). A threshold of 36 cycles was used and *p*-values adjusted using the Benjamini–Hochberg method. Primer sequences and amplification conditions can be found in Tables S5 and S6. If qPCR failed because of low expression or similarity to paralogs preventing gene specific primer design, the next DEG in the list was assayed until ten were successfully assayed for each category (File S1).

### 4.5. DE Gene Analysis

To analyze relationships between DEGs, STRING (v11.0) was used to generate association networks using a low interaction score (0.100), and the *A. thaliana* database [58]. Pistil and stamen DEG data sets were combined separately to generate tissue specific networks. Networks were uploaded to the Network Data Exchange (NDEx) repository [83] for public access (UUIDs are listed in Table S3). Networks were clustered using NCMine (v1.3.0), which uses a modified MCODE algorithm for community detection, to identify functional subnetworks [59]. Pistil and stamen networks were compared using DyNet (v1.0.0) using default settings [84]. STRING generated networks were analyzed with Cytoscape (v3.7.1) coupled with ClueGO (v2.54), to perform more rigorous GO enrichment analysis and create GO clusters [85,86]. Genes not mapped in STRING were included for ClueGO analysis. *A. thaliana* GO, KEGG, and Reactome databases were used as lines of evidence. ClueGo parameters used were, GO term levels of 0–20, kappa score of 0.400, and adjusted *p*-value score of < 0.05 for individual terms. *p*-values were adjusted using the Bonferroni’s step-down method.

### 4.6. KEGG Analysis

KofamKOALA was used to assign KEGG orthology identifiers and identify associated KEGG pathways using FASTA files generated through STRING [87]. Only significant annotations (*p* < 0.05) were used in reconstruction of KEGG pathways.

## Data Availability

The RNA-seq data and annotated transcriptome have been submitted to NCBI Bioproject PRJNA589060. STRING Networks are available from the Network Data Exchange (NDEx) repository, UUIDs can be found in Table S4).

## Supplementary Materials

The following are available online at https://www.mdpi.com/2223-7747/9/6/713/s1. Table S1: Resulting clean RNA-Seq reads as provided by NovoGene. Table S2: GO terms associated with TsYUC6 in stamens. Table S3: GO terms identified in the reciprocal dataset. Table S4: NDEx UUIDs for STRING generated pathways. Table S5: Primers for RT-qPCR; Table S6: ABI fast 7500 conditions. Figure S1: MA plots and heatmaps of DEGs in the four tissue types. Figure S2: Enriched GO terms. Figure S3: Depleted GO terms. Figure S4: KEGG analysis of *Turnera subulata*; STRING networks. Figure S5: DEGs in pistils. Figure S6: DEGs in stamens. Figure S7: First neighbors of BAHD. Figure S8: BAHD Cluster 8. Figure S9: First neighbors of YUC6. Figure S10: YUC6 Cluster 4. Figure S11: YUC6 Cluster 5. Figure S12: YUC6 Cluster 6. Figure S13: YUC6 Cluster 13. Figure S14: First neighbors of SPH1. Figure S15: SPH1 Cluster 1. File S1: Descriptions of these DEGs, read count tables, Arabidopsis homologs for each differential dataset. File S2: Comprehensive lists of the clustered enriched and depleted GO terms. File S3: Comprehensive list of Enriched and depleted pathways between samples types. File S4: Comprehensive lists of genes in predicted associations identified in STRING analyses.
