# Supplementary material for: Transcriptome and Network Analyses of Heterostyly in Turnera subulata Provide Mechanistic Insights: Are S-Loci a Red-Light for Pistil Elongation?
Source: Plants (Basel). 2020 Jun 3;9(6):713. doi: 10.3390/plants9060713 (PMC7356734; doi:10.3390/plants9060713)
Supplement: Supplementary file 1 [file plants-09-00713-s001.zip › plants-804445-XML01-supplementary/plants-804445_Tables S1-S6 and Figures S1-S15.docx]

**Table S1.** Resulting clean RNA-Seq reads as provided by NovoGene.

| Sample | Total clean reads of each biological replicate | | Percentage of reads > Q30 |
| --- | --- | --- | --- |
| Mature S-morph stamen *T. subulata* | MSS1 | 21985173 | 93.80% |
|  | MSS2 | 21410244 | 93.66% |
|  | MSS3 | 19259839 | 93.34% |
| Mature L-morph stamen *T. subulata* | MLS1 | 20014557 | 93.22% |
|  | MLS2 | 20992192 | 93.60% |
|  | MLS3 | 19513504 | 93.27% |
| Mature S-morph pistil *T. subulata* | MSP1 | 21013901 | 94.03% |
|  | MSP2 | 21270843 | 92.43% |
|  | MSP3 | 23289016 | 92.54% |
| Mature L-morph pistil *T. subulata* | MLP1 | 22204429 | 93.66% |
|  | MLP2 | 20540787 | 93.40% |
|  | MLP3 | 20229819 | 93.68% |
| Young S-morph stamen *T. subulata* | YSS1 | 22040798 | 90.47% |
|  | YSS2 | 18710569 | 90.28% |
|  | YSS3 | 25145677 | 89.70% |
| Young L-morph stamen *T. subulata* | YLS1 | 21361832 | 91.00% |
|  | YLS2 | 20279416 | 90.50% |
|  | YLS3 | 25514716 | 90.77% |
| Young S-morph pistil *T. subulata* | YSP1 | 24055639 | 93.28% |
|  | YSP2 | 23861671 | 93.86% |
|  | YSP3 | 35167357 | 93.61% |
| Young L-morph pistil *T. subulata* | YLP1 | 20432172 | 93.60% |
|  | YLP2 | 19302178 | 93.82% |
|  | YLP3 | 19897212 | 92.69% |
| Mature S-morph bud *T. joelii* | TjMSB1 | 25329559 | 92.07% |
|  | TjMSB2 | 20763757 | 92.29% |
|  | TjMSB3 | 21419929 | 91.70% |
| Mature L-morph bud *T. joelii* | TjMLB1 | 20665430 | 92.44% |
|  | TjMLB2 | 19673876 | 92.44% |
|  | TjMLB3 | 21504784 | 92.11% |
| Young S-morph bud *T. joelii* | TjYSB1 | 42262170 | 92.04% |
|  | TjYSB2 | 18563409 | 92.34% |
|  | TjYSB3 | 19833947 | 91.67% |
| Young L-morph bud *T. joelii* | TjYLB1 | 20565535 | 91.39% |
|  | TjYLB2 | 19969227 | 91.15% |
|  | TjYLB3 | 21922213 | 90.85% |

Total clean RNA sequence reads after processing by NovoGene. All clean reads had a minimum quality score (Q) of 30. Clean reads were later used in calculating transcript abundance and later differential gene expression analysis.

**Table S2.** GO terms associated with TsYUC6 in young stamens.

| **GO:0010035** | **response to inorganic substance** |
| --- | --- |
| GO:0006979 | response to oxidative stress |
| GO:0005622 | intracellular |
| GO:0044424 | intracellular part |
| GO:0016705 | oxidoreductase activity, acting on paired donors, with incorporation or reduction of molecular oxygen |
| GO:0016709 | oxidoreductase activity, acting on paired donors, with incorporation or reduction of molecular oxygen, NAD(P)H as one donor, and incorporation of one atom of oxygen |
| GO:0005618 | cell wall |

We identified seven enriched GO terms in our young stamen dataset that associated with the *A. thaliana* homolog of *TsYUC6*. As they are enriched in the dataset and associated with *AtYUCCA6*, they have the potential to represent biological functions that are important for the presence of distyly.

**Table S3.** GO terms identified in the reciprocal dataset.

| **GO cluster** | **GOID** | **GOTerm** | **Genes** |
| --- | --- | --- | --- |
| catalytic activity | GO:0003824 | catalytic activity | 37 |
| nucleus | GO:0005634 | nucleus | 6 |
| cell wall organization | GO:0045229 | external encapsulating structure organization | 8 |
|  | GO:0071554 | cell wall organization or biogenesis | 8 |
|  | GO:0071555 | cell wall organization | 8 |
| terpenoid biosynthetic process | GO:0006720 | isoprenoid metabolic process | 5 |
|  | GO:0006721 | terpenoid metabolic process | 5 |
|  | GO:0008299 | isoprenoid biosynthetic process | 5 |
|  | GO:0016114 | terpenoid biosynthetic process | 5 |
|  | GO:0042299 | lupeol synthase activity | 2 |
|  | GO:0044255 | cellular lipid metabolic process | 8 |
| intrinsic component of membrane | GO:0005215 | transporter activity | 13 |
|  | GO:0006855 | drug transmembrane transport | 3 |
|  | GO:0016020 | membrane | 32 |
|  | GO:0016021 | integral component of membrane | 26 |
|  | GO:0016820 | hydrolase activity, acting on acid anhydrides, catalyzing transmembrane movement of substances | 5 |
|  | GO:0022804 | active transmembrane transporter activity | 8 |
|  | GO:0022857 | transmembrane transporter activity | 11 |
|  | GO:0031224 | intrinsic component of membrane | 27 |
|  | GO:0042626 | ATPase activity, coupled to transmembrane movement of substances | 5 |
|  | GO:0043492 | ATPase activity, coupled to movement of substances | 5 |
|  | GO:0044425 | membrane part | 28 |
|  | GO:0071944 | cell periphery | 21 |

Genes that were reciprocally expressed between the pistil and stamen of *T. subulata* had the potential to be important for SI. Reciprocally expressed for this analysis was defined as an enrichment of a transcript in one organ and the depletion of the same transcript in the other organ. Transcripts were only considered reciprocal if the differences were between the same developmental stages. Meaning, a transcript that differed in expression between a mature and young tissue were not considered reciprocally expressed. While several GO terms were identified as overrepresented in this dataset, no clear conclusions related to SI were drawn from this data.

**Table S4.** NDEx UUIDs for STRING generated pathways.

| **Network** | **UUID** |
| --- | --- |
| Pistil | 8c960833-f085-11e9-bb65-0ac135e8bacf |
| Stamen | b8bf1d50-dbca-11e9-bb65-0ac135e8bacf |
| Reciprocal | 95319266-dbdd-11e9-bb65-0ac135e8bacf |
| BAHD Cluster (1) | e06a9891-dbd5-11e9-bb65-0ac135e8bacf |
| BAHD Cluster (2) | fba3e536-dbd5-11e9-bb65-0ac135e8bacf |
| BAHD Cluster (3) | 3c406e16-f086-11e9-bb65-0ac135e8bacf |
| BAHD Cluster (4) | 46de0538-f086-11e9-bb65-0ac135e8bacf |
| BAHD Cluster (5) | 5312b71a-f086-11e9-bb65-0ac135e8bacf |
| BAHD Cluster (6) | f6138765-dbd6-11e9-bb65-0ac135e8bacf |
| BAHD Cluster (7) | 5c62235c-f086-11e9-bb65-0ac135e8bacf |
| BAHD Cluster (8) | 64684f80-dbd7-11e9-bb65-0ac135e8bacf |
| BAHD first neighbors | 8bffc63f-f086-11e9-bb65-0ac135e8bacf |
| SPH1 Cluster (1) | 9ca595ae-dbe2-11e9-bb65-0ac135e8bacf |
| SPH1 first neighbors | 0d6be26d-dbd2-11e9-bb65-0ac135e8bacf |
| YUCCA6 Cluster (1) | f6e9eb9b-dbcb-11e9-bb65-0ac135e8bacf |
| YUCCA6 Cluster (2) | 6ad3457a-dbcd-11e9-bb65-0ac135e8bacf |
| YUCCA6 Cluster (3) | ca41a93d-dbcd-11e9-bb65-0ac135e8bacf |
| YUCCA6 Cluster (4) | f6183201-dbcd-11e9-bb65-0ac135e8bacf |
| YUCCA6 Cluster (5) | 1c800b24-dbce-11e9-bb65-0ac135e8bacf |
| YUCCA6 Cluster (6) | 5c403be9-dbce-11e9-bb65-0ac135e8bacf |
| YUCCA6 Cluster (7) | a8e13d1c-dbce-11e9-bb65-0ac135e8bacf |
| YUCCA6 Cluster (8) | d2d59861-dbce-11e9-bb65-0ac135e8bacf |
| YUCCA6 Cluster (9) | 01c65976-dbcf-11e9-bb65-0ac135e8bacf |
| YUCCA6 Cluster (10) | 29847454-dbe9-11e9-bb65-0ac135e8bacf |
| YUCCA6 Cluster (11) | 47a6fe36-dbe9-11e9-bb65-0ac135e8bacf |
| YUCCA6 Cluster (12) | 7b358a5a-dbe9-11e9-bb65-0ac135e8bacf |
| YUCCA6 Cluster (13) | a40366fc-dbe9-11e9-bb65-0ac135e8bacf |
| YUCCA6 first neighbors | 6b38e796-dbd1-11e9-bb65-0ac135e8bacf |

Networks containing genes differentially expressed (SvL) in the pistil and stamen of *T. subulata* were generated using *A. thaliana* homologs and STRING [1]. Networks containing the predicted first neighbors of the three S-locus genes (*TsBAHD, TsSPH1,* and *TsYUC6*) were identified. Clusters containing the three S-locus genes were identified using NCMine (Tadaka and Kinoshita, 2016). As the networks are vast and contain hundreds of genes and are much easier viewed as dynamic graphs than static images, all networks were uploaded to NDEx [3]. To find a given network, reads just need to access the NDEx website and search with the given UUID.

**Table S5.** Primers for RT-qPCR.

| **Gene ID** | **Forward Primer 5' to 3'** | **Reverse Primer 5' to 3'** |
| --- | --- | --- |
| Tsub_00001208-RA | AGCAACAAGAAGGGCCA | CACGAGCAATGAGGTCATC |
| Tsub_00001633-RA | AATCGGAGCAGAGATGCA | ACTAGGATATTGAGCTTGCCATC |
| Tsub_00001794-RA | AGCTAGGACGAGACTCACCA | AGGCGCCATGATCTGTAG |
| Tsub_00002369-RA | CAATCCAATGCTTGCCTC | CTTCTCCGCCAACCACT |
| Tsub_00002485-RA | CAAGAGGCGTTTGATGATG | ACTGATCCTCCCAGCTCTATCT |
| Tsub_00002929-RA | TCAATCCATTACAGGAATGACA | GTACACTCCCAACACAATTG |
| Tsub_00003538-RA | ACCAGAAGGACTTGTGGGA | TCCTCAGTTTGATCATCTTGTTC |
| Tsub_00004389-RA | CTCAGACTGGCTCGCTTC | ACCGTATTGGGCAGTTATCA |
| Tsub_00004611-RA | TGCCCCATGTAGCATTG | GAGGTTACTCAATTTGCGGA |
| Tsub_00004975-RA | AGTTCCATCCGATGCAGTAG | TCCACATTTCCTTCGCTC |
| Tsub_00005197-RA | ACACCCAATGCAGTCCA | GAATAAAGCAACCATCAAGCA |
| Tsub_00005588-RA | ACGCAACATGCTTGATTGT | GACCTACGACCTGATCAATTGT |
| Tsub_00005883-RA | AGGAGGAGCGCTAGCATG | TGAGCATGACTAGCAACCTGA |
| Tsub_00005928-RA | TGGCTGCAGCTTGTCTTC | ACTGCTGGTGCCCAAGTAG |
| Tsub_00006584-RA | CACATTTTGCTGTTCCACAC | ACTACGCCAGGGTTGACA |
| Tsub_00006790-RA | TCGGTGTTCATTTCCTTCA | CTTGGCGTTTTGAAGCTCT |
| Tsub_00006885-RA | AGGACATAGATGCCACCAAG | CTCCATTAAGCCAGAGAAGAAGT |
| Tsub_00007006-RA | TGGAAATGAAAGGTCCCTG | TCTTTGGAAGTCACATCCTTG |
| Tsub_00007139-RA | TGGTGAAATGGCAGAAAGA | TGTGATGACCTCTGGGGT |
| Tsub_00008119-RA | AGCAATCATGGCCGAGT | GTGACAGCCGGGTTGA |
| Tsub_00008428-RA | GATAAGGCCCATGTTAGATTCA | CACGAGTTGAATGCATTCTTC |
| Tsub_00010469-RA | TGAGATCACCAGGGGAGA | ACTTGGTTGAGCATAAGCCTC |
| Tsub_00010651-RA | ACCAACAGATTCCAGGACCT | AGAGACGCGTCCAGCA |
| Tsub_00010763-RA | CAGCTTCGTGGATCCAGT | TGGAGGAGAAGGCCATC |
| Tsub_00010944-RA | CAGTAACCTGCAGGATTTCTAGTC | CTAGCTGCTTCCTCACAATCTC |
| Tsub_00011283-RA | ACACCAGGTCCAAGTCCA | GAGGAGGAACACCGGAGT |
| Tsub_00011790-RA | TGCTAATGGAGGGAACCA | TGCATGTGATGATGACAATG |
| Tsub_00011964-RA | CAAAGCCTTTGTATGATTCCA | AGGAGGTATTGCATTCCGTAG |
| Tsub_00011966-RA | CAAGGAATGAGCAAATGGA | AGGCTCCACTGATTACCTCA |
| Tsub_00012862-RA | GTAGTGAGGGATGGTGGAGA | CTGTCATGAATGCTCCCTG |
| Tsub_00013327-RA | CAGATGTCTGCAACCGTTC | GAATGGCACTGTGGGTGT |
| Tsub_00013876-RA | ACAATGACTGCCAGCAGAAC | GTTCCAATTACGCTCCTGGT |
| Tsub_00013950-RA | GATTGCCATCATCGGGT | AGAGGATGGAGGTGCAGAG |
| Tsub_00014240-RA | CTAACAAGCCTGCAGTTCCA | GACAAGAGCGGAGGAGGT |
| Tsub_00014550-RA | CAACCTCAGCTATTCTCCCA | ACCCCAGGTTTGCAATCT |
| Tsub_00014629-RA | GAGTTGCAGGCCAACAAG | CTCCTCCATCTCCACACCT |
| Tsub_00014781-RA | AGACACCCCAGTTTCAGTTTC | ACATACCCCTTGAACTCCTCTC |
| Tsub_00014844-RA | CTGGTGAAACGGTTGGAG | CATCCACGGCTTTGTCA |
| Tsub_00015101-RA | TCGAGCATCAAGAGTGGAG | CATTGCCCTCACTGTCTTG |
| Tsub_00015820-RA | TCATCATCAACAAGAACCGAG | ACTGGTCGAGGTACCTCAAGA |
| Tsub_00016130-RA | AGCTGATCAATGCTGCTAAGA | CACTCGAGCTGCCCAC |
| Tsub_00016284-RA | CAGTGGGTGAGTGGATAGTAGTG | GCGAAGAGAGAGTTGGATCAC |
| Tsub_00016976-RA | GAGGAGGAAAAAGTCCGAAG | CTTCCGGGTCCTCAAAGT |
| Tsub_00017134-RA | CTTCCTGCTTCACTCCCA | CACGATGATGGTGGCA |
| Tsub_00017479-RA | AGCTTAGCATTGCAGCAGA | CACAATCCTCGCGGAC |
| Tsub_00018041-RA | AGCAGGACATCATGGCTAGT | TCCCATCCTCGTAGAATTCA |
| Tsub_00018140-RA | TGGACGCAGAGTCTGAAAG | AGTGGTTCATTCCGTGGTAG |
| Tsub_00018628-RA | CATTCCTGATCCTGAAGACATAC | TGAAGGTGCTCCCCAGT |
| Tsub_00018986-RA | AGAGATCAGTAGCAAAGCTGGA | TCATTCCCAGAGTGTTCTTCA |
| Tsub_00019225-RA | GACATGGATGACATTCTTGATG | ACCACTCTGTCTATTTCCTCATGT |
| Tsub_00019871-RA | TCAAACCAATGTTACTGGCA | TGGTATCACTGCACCCACT |
| Tsub_00019919-RA | GAGCCATGGCAACGTTAG | CATCCATTGCTTACCCAGAG |
| Tsub_00020077-RA | CTAAGGCCAGTCTCCATCTG | AGAAGCTCTCCATGTCATCCT |
| Tsub_00020222-RA | GAGAGGCAGAGGTGCAAG | GAATGAACAGATACCCACCCT |
| Tsub_00020463-RA | GATGTAGGCAGCCCATTG | CTCGCAAATTCTCTCAATCTCT |
| Tsub_00021189-RA | TGTGGTGATTATGGCTGCT | ACCAGTAGCCCAATATGCCT |
| Tsub_00021280-RA | TGAACTCAAGGCCTTTGAAG | TCTCAACCATATCTTGCTCTGTC |
| Tsub_00021297-RA | AGTGGATAGTCCTCGTGAGAGA | ACCAACGTGACACAGCAGT |
| Tsub_00021856-RA | AGTACCGGCAGCAGTGAG | TGTGAGTCAGCCGCCT |
| Tsub_00022067-RA | GAATCTGACACCACCCCTC | TCAGTTTGAAACTTGGGCA |
| Tsub_00022248-RA | ACATGTTAGGCAACCTTCCA | ACCGTAGGACATGTACAAGGAG |
| Tsub_00022885-RA | CACAACCCCTGTCACACA | AGCACCTGCATTAGTCCAAC |
| Tsub_00023348-RA | AGTAGCAGCACCCGCA | TGTTTTCCGGTGCTGC |
| Tsub_00023775-RA | AGCCTCAATTGTGACATCAAG | TAAGGAGGTGGCACATTCA |
| Tsub_00025053-RA | GAGATCGAACAGTGCCAGA | AGGCACTCTTCGAATCGTC |
| Tsub_00025930-RA | TGGTGTCCATTCAATCCAC | TGGCAAACTTTCTTGGGA |
| Tsub_00026159-RA | TCAGGATGATTGATGAGCTTC | TGCCAAGGAAGGAAGTCA |
| Tsub_00026848-RA | GTTGCTGTGTGCGGTTC | ACTTCAGAAATCCACATGGTATG |
| Tsub_00026881-RA | TGACCTTGTGGAAAGCTGA | ACAGGCCTTCATGATATCCA |
| Tsub_00027977-RA | TGGAACCCTAAGAGGTTGTG | CTTCTTGAGGTTTTGGAGCA |
| Tsub_00028003-RA | ACCTGAAGATGAGAATGACTGCT | TCCATGATATCAGCAAGGACA |
| Tsub_00028004-RA | GAATTGGGCTCTGGAGGT | CAACTTTGAAGAAGCTCCCTC |
| Tsub_00029014-RA | CATGAAGAGGGACATTCTTGA | AGCCACAGGGGAGATGA |
| Tsub_00029694-RA | TCCCAAAGGTGATGGCT | TCTCGATTGATGAACGCTC |
| Tsub_00029758-RA | GTGGAGACTGTGACGGAGA | CTTCATGTGCCTTCCCAG |
| Tsub_00030224-RA | GACAGAGTATCCGAACCTGGT | ACAGCGAAACGAGCGA |
| Tsub_00030305-RA | CTGAGACTTTCGAGCTGTACAAG | CTTCCACAAGCTTGGCTCT |
| UEV1D | AGCCAGCCAAGTCGTCT | CATCGTCCATTCCATAGCTTAC |
| β Tubulin | AGATTTTCCGACCCGACA | GCAATCACAATTCTCGGCT |

**Table 6.** ABI fast 7500 conditions.

| **Holding Stage** | **95.0 ^o^C** | **10:00** |
| --- | --- | --- |
| Cycling Stage (40 cycles) | 95.0 ^o^C  61.5 ^o^C | 00:15  00:30 |
| Melt Curve Stage  Step 1  Step 2  Step 3  Step 4 | 95.0 ^o^C  100%  61.5 ^o^C  1%  95.0 ^o^C  100%  61.5 ^o^C | 00:15  01:00  00:15  00:15 |

Modified conditions used for all RT-qPCR runs.

**
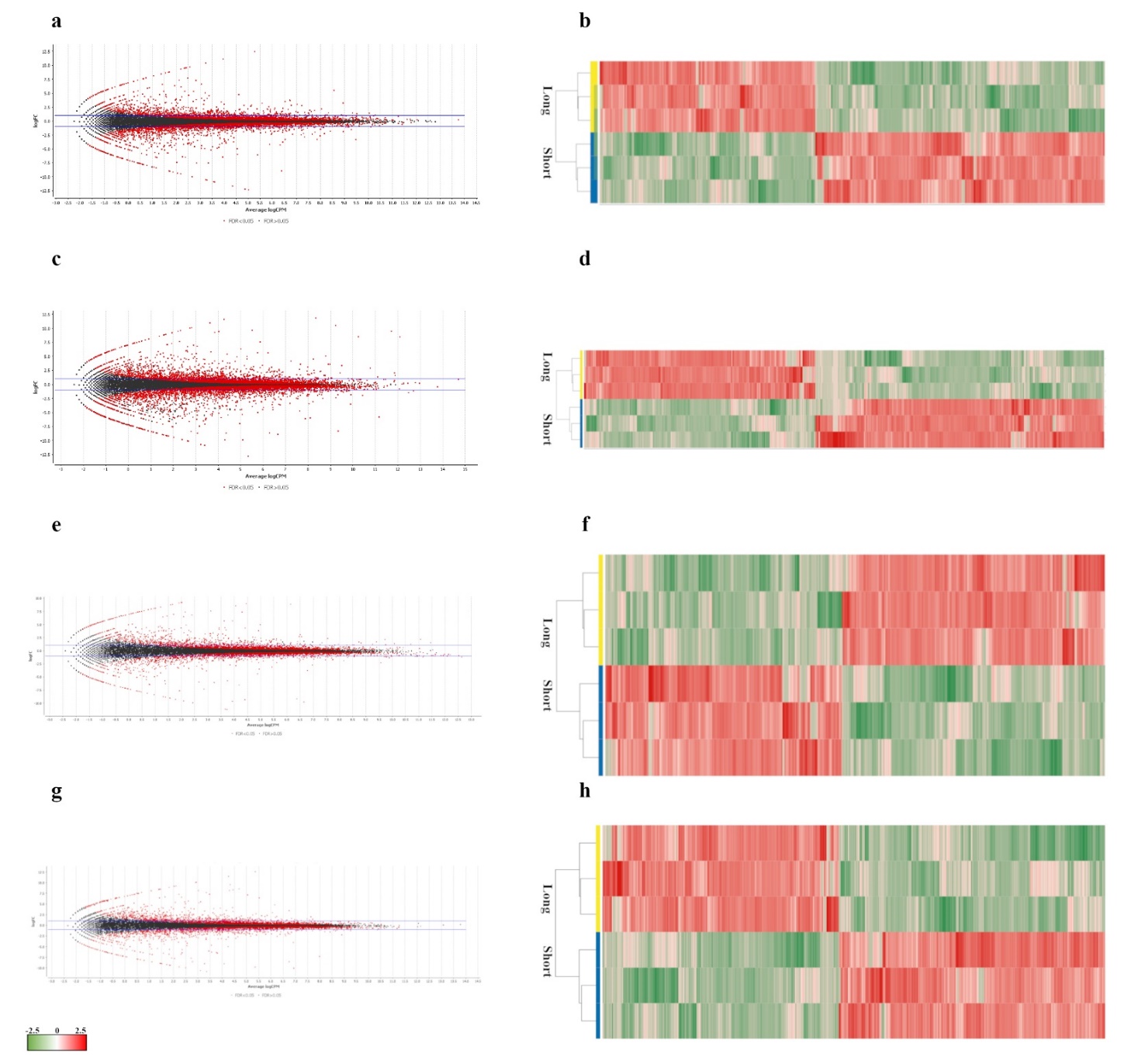
**

**Figure S1.** MA plots and heatmaps of DEGs in the four tissue types. Differentially expressed genes (SvL) within different tissue types. MA plots of DEGs in mature stamen (a), mature pistil (c), young stamen (e), and young pistil (g). MA plots were included to quickly represent the differences in gene expression between the S- and L-morphs and to show that data was properly normalized prior to further analysis. Heatmaps of DEGs in mature stamen (b), mature pistil (d), young stamen (f), and young pistil (h). Heatmaps were included to quickly show readers the ratio of enriched and depleted genes in the S- and L-morphs. An adjusted p-value of < 0.05 and |Log_2_(FC)|>1.0 were used when generating both graphs. Heatmaps were generated using InCHlib [4].

**
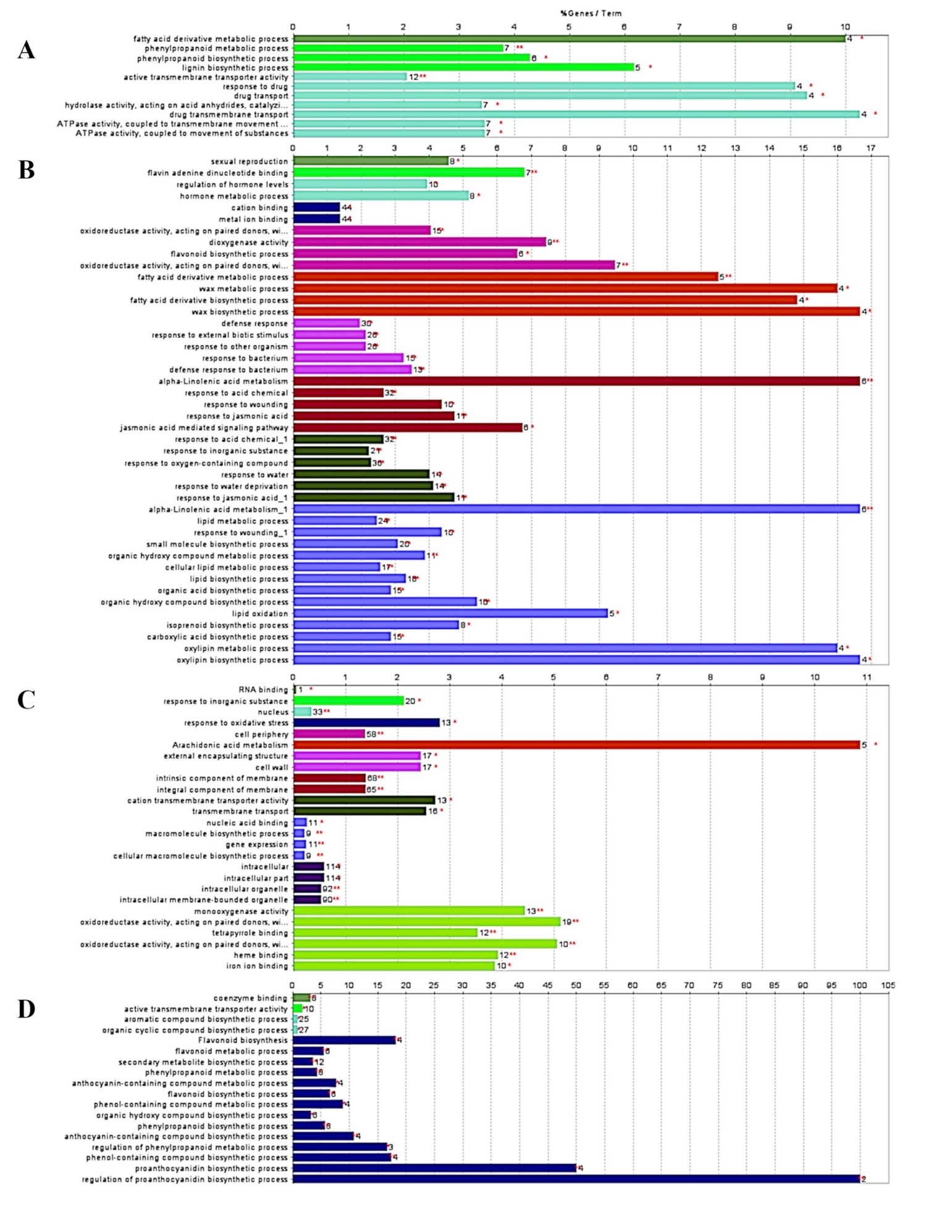
**

**Figure 2. Enriched GO terms.** ClueGO generated graphs for enriched (SvL) GO terms found in the mature stamen (a), mature pistil (b), young stamen (c), and young pistil (d). GO terms provide a quick look at biological, cellular, and molecular processes that may be important to the system based on the presence of genes related to these processes. Bars are colored to represent the cluster the term belongs to. %Gene/term represents the genes in our dataset associated with a given term / all genes associated with a given term in A. thaliana. Numbers next to each bar represents the number of genes associated with a given term. Each term has a p-value < 0.05.

**
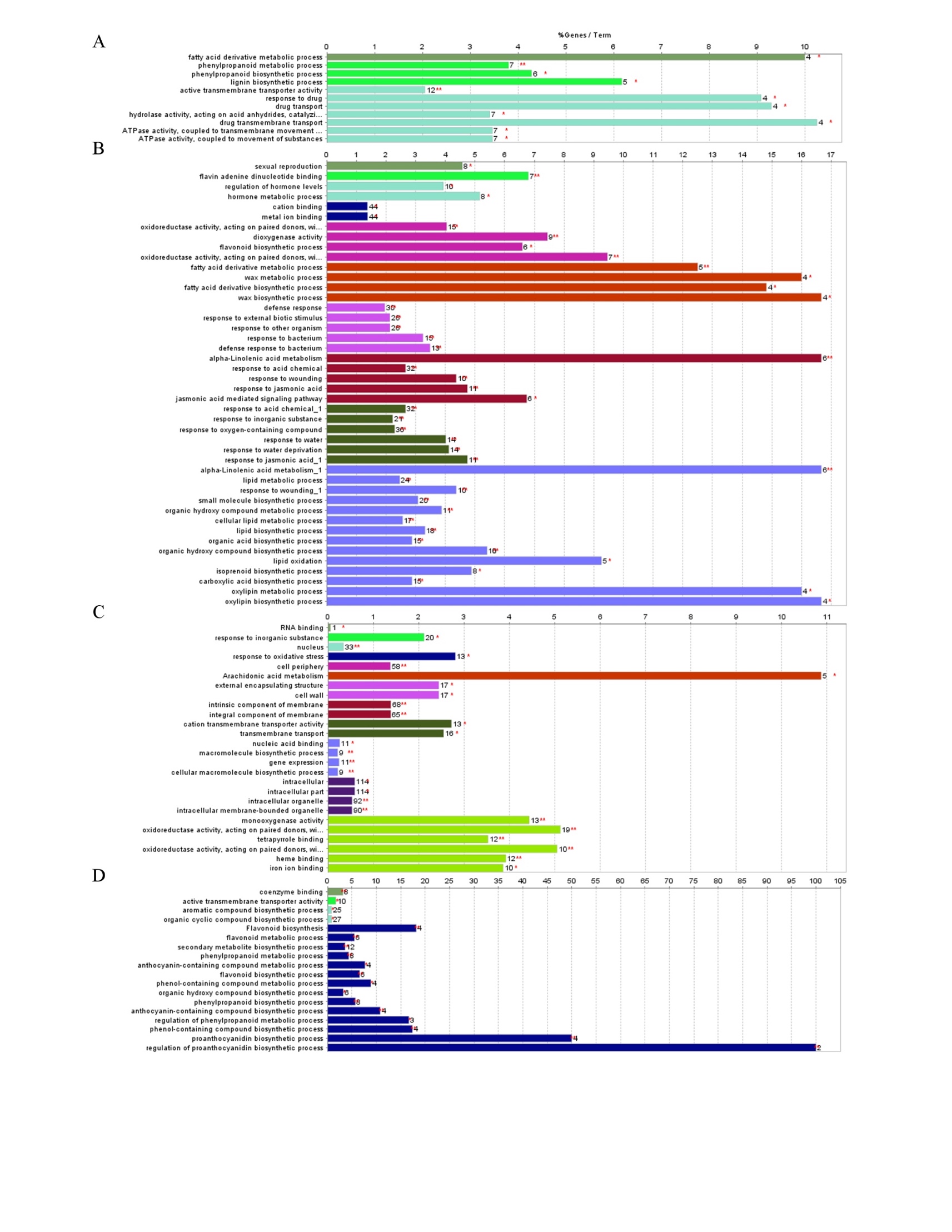
**

**Figure S3.** Depleted GO terms. ClueGO generated graphs for depleted (SvL) GO terms found in the mature stamen (a), mature pistil (b), young stamen (c), and young pistil (d). GO terms provide a quick look at biological, cellular, and molecular processes that may be important to the system based on the presence of genes related to these processes. Bars are colored to represent the cluster a given term is a member of. %Gene/term represents the genes in our dataset associated with a given term / all genes associated with a given term in A. thaliana. Numbers next to each bar represents the number of genes associated with a given term. Each term has a p-value < 0.05.


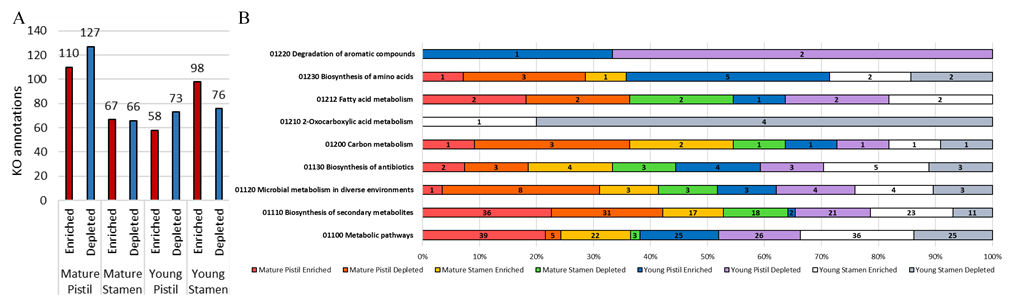


**Figure S4. KEGG analysis of *Turnera subulata.*** To identify potential reaction networks of importance, we used our *A. thaliana* homolog datasets with KofamKOALA to identify enriched and depleted (SvL) KEGG pathways [5]. A total of 675 KO annotations were identified in our eight datasets (a). KEGG overview maps are composed of several smaller pathways that are all related to one general purpose. These allow for a quick interpretation of KO pathway enrichment and depletion (b). From this analysis, changes to metabolic pathways and biosynthesis of secondary metabolites are likely important for the presence of distyly in both female and male aspects during the different stages of development.


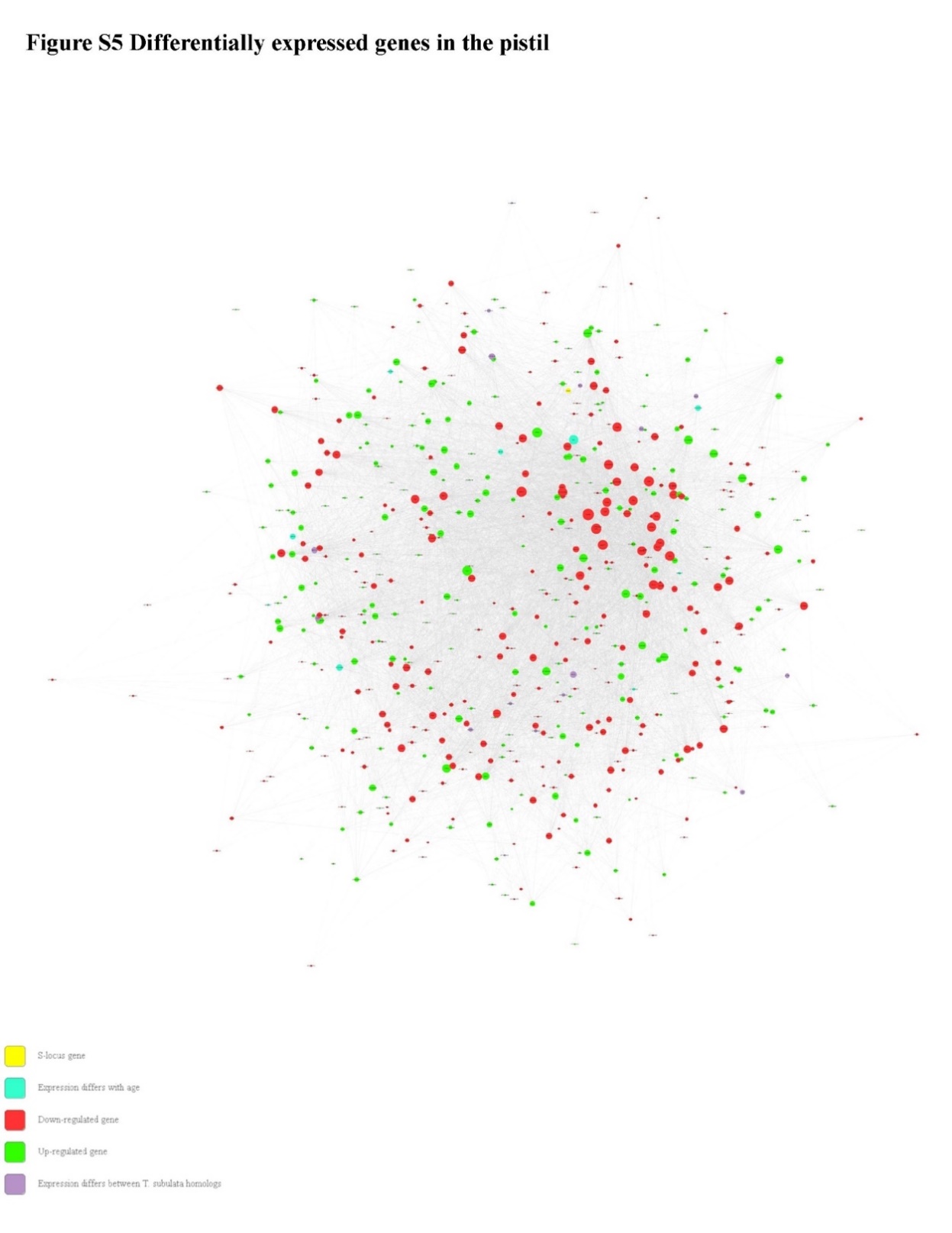


**Figure S5. Differentially expressed genes in the pistil.** STRING generated network of the DEGs in the pistils of Turnera subulata [1]. This network contains genes from both stages of development as changes in expression of a gene in early development may affect expression of a gene later in development. By generating this network, we hoped to find subnetworks related to the female characteristics of heterostyly in T. subulata. This network has been uploaded to the Network Data Exchange (NDEx) repository, UUID can be found in table S4.

**
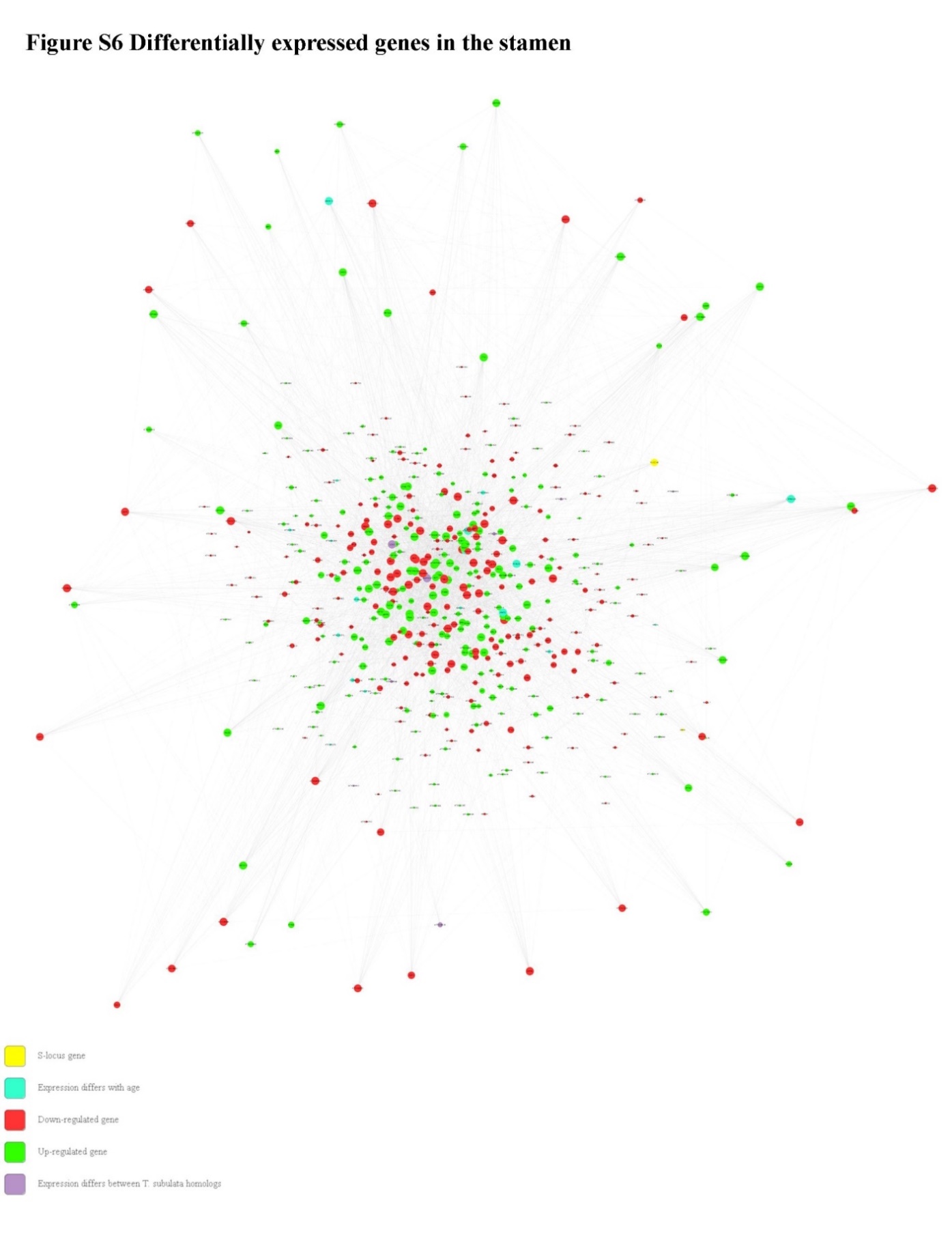
**

**Figure S6. Differentially expressed genes in the stamen.** STRING generated network of the DEGs in the stamens of Turnera subulata [1]. This network contains genes from both stages of development as changes to the expression of a gene in early development may affect expression of a gene later in development. By generating this network, we hoped to find subnetworks related to the male characteristics of heterostyly in T. subulata. This network has been uploaded to the Network Data Exchange (NDEx) repository, UUID can be found in table S4.

**
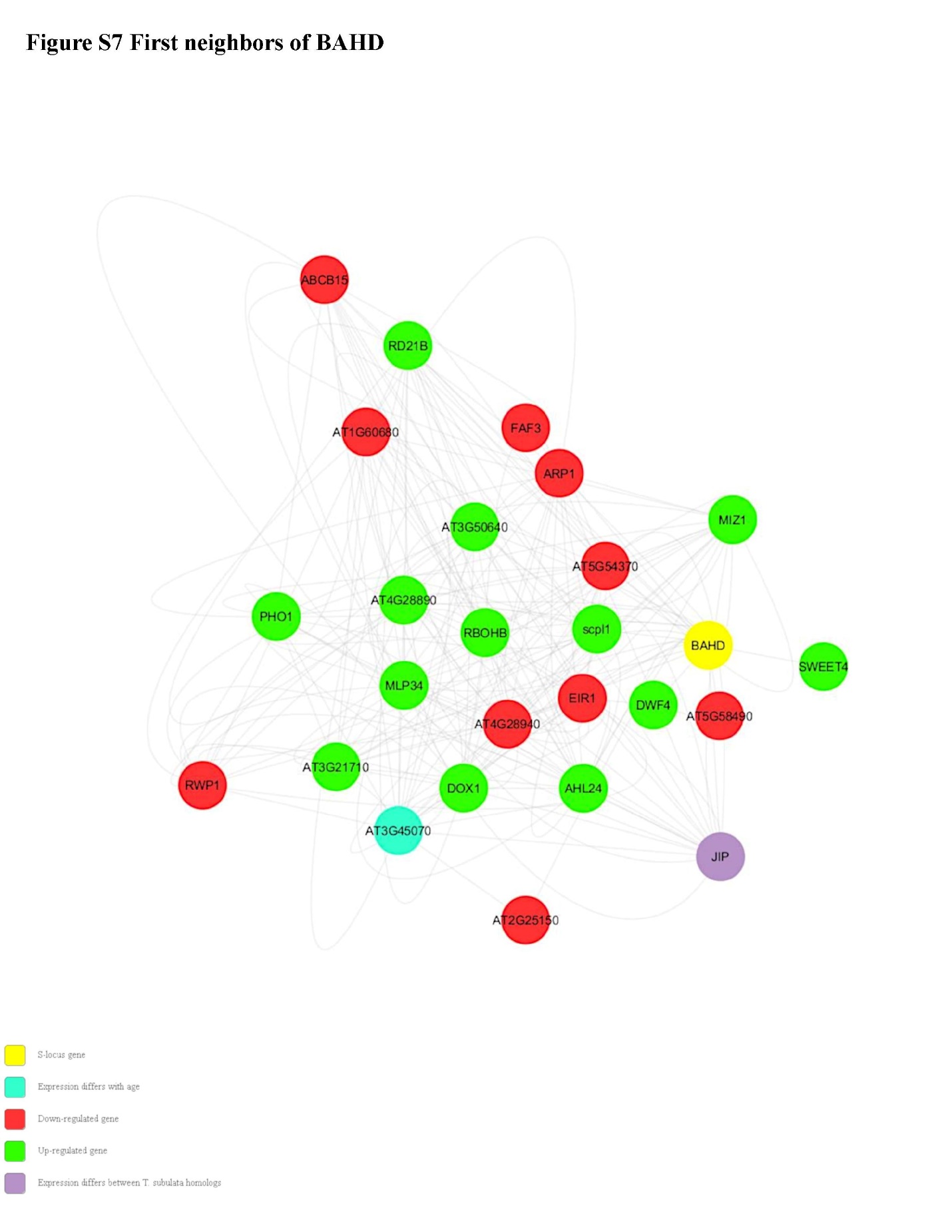
**

**Figure S7. First neighbors of BAHD.** To identify genes that TsBAHD may be directly influencing, we identified all first neighbors of BAHD, (genes predicted to directly associate with the A. thaliana homolog of TsBAHD). 25 genes were first neighbors of BAHD. Several of these first neighbors are genes related to brassinosteroid (BR). Changes inexpression of BR related genes supports the hypothesis that TsBAHD activity leads to changes to BR levels. This identification of the first neighbors of BAHD allowed for identification of genes that may be of interest for future studies.

**
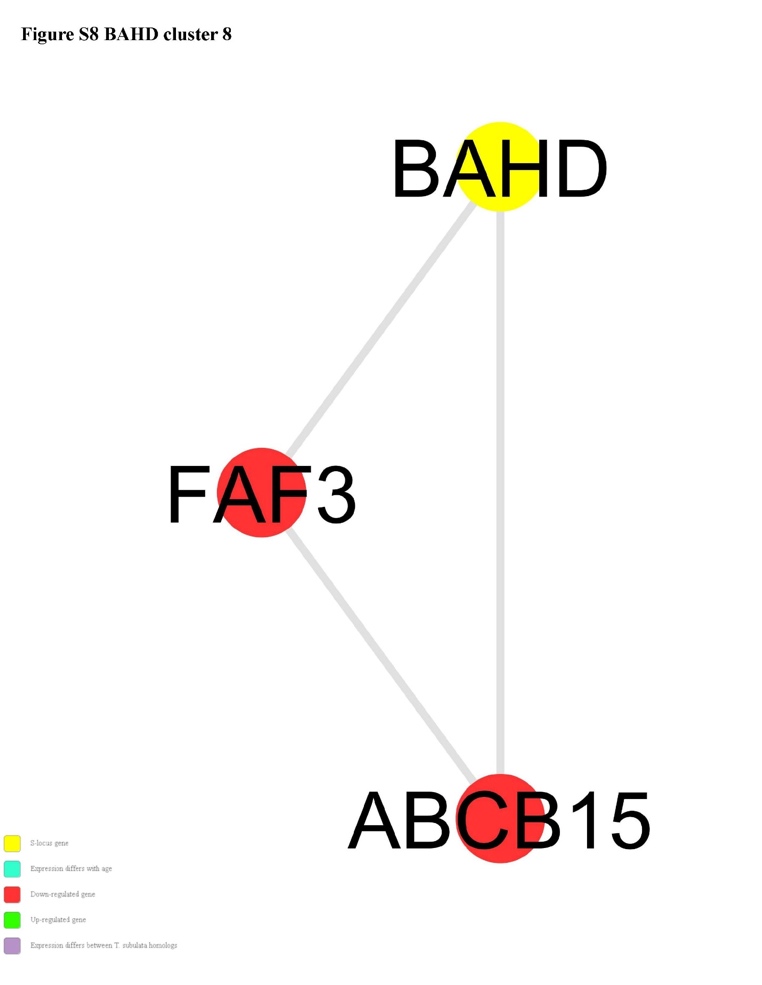
**

**Figure S8. BAHD cluster 8.** To identify genes related to brassinosteroids we used NCMine to generate clusters from our pistil network and isolated those containing BAHD [2]. Eight clusters were identified that contained BAHD. All BAHD containing clusters can be found on NDEx (table S4). Cluster 8 has been included due to its high clustering coefficient (1.0) and low heterogeneity score (0.0).


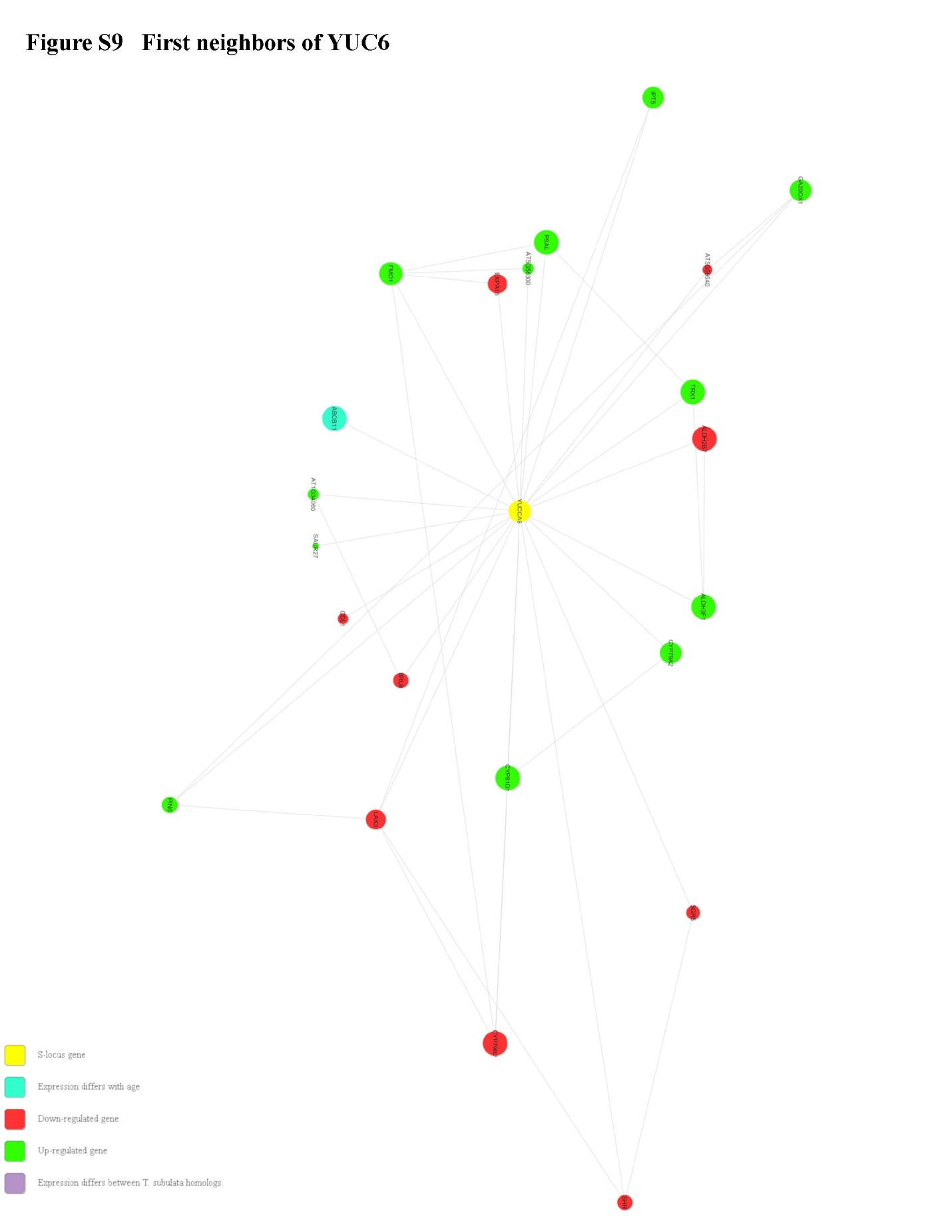


**Figure S9. First neighbors of Yuc6.** YUC6 had 22 first neighbors in our stamen DEG dataset, several of which were related to auxin response, synthesis, or transport. First neighbors are genes predicted to associate directly with the gene of interest; thus, by identifying first neighbors, we can identify genes of interest for future study.


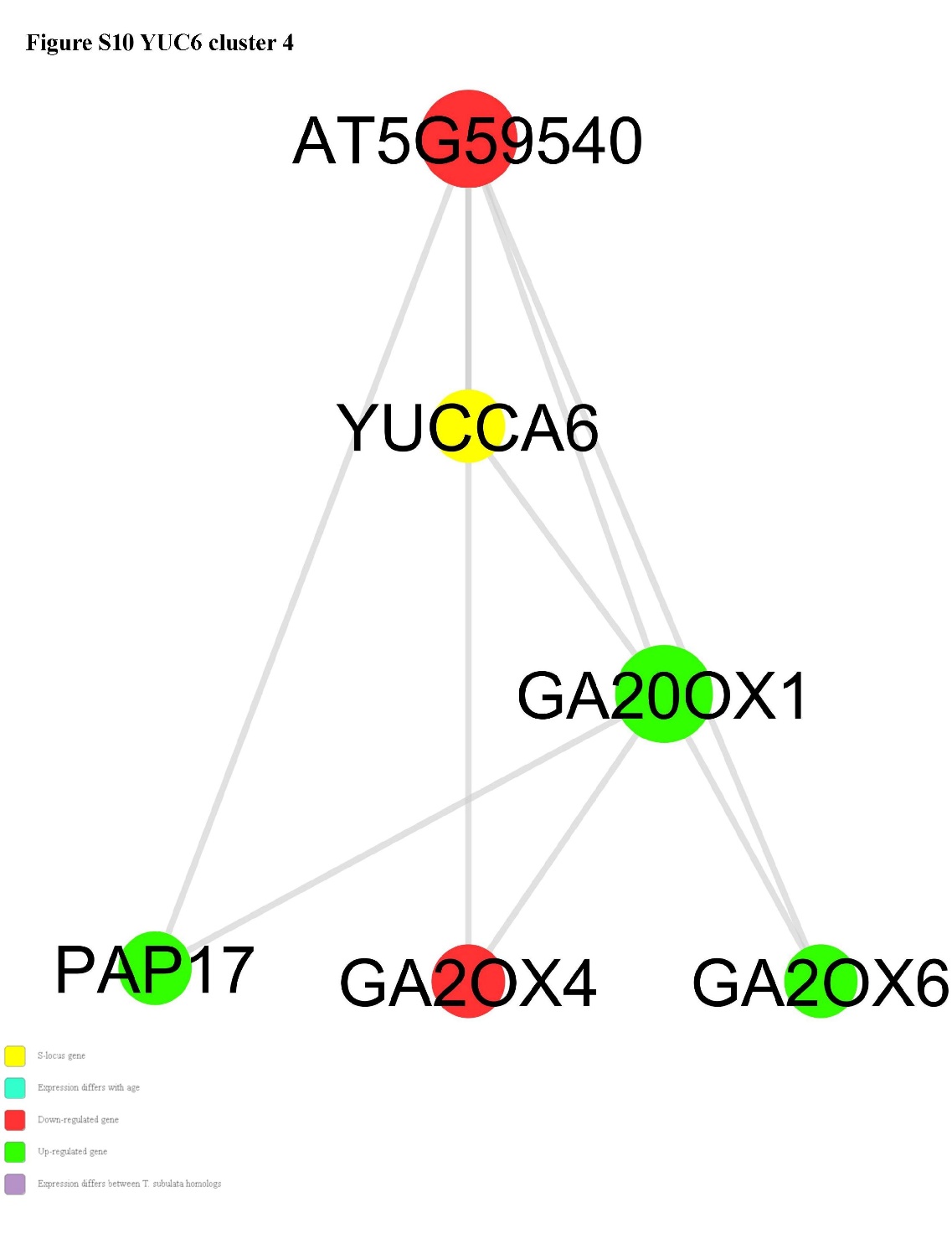


**Figure 10. YUC6 cluster 4.** To identify additional auxin related genes in our stamen DEG dataset, we generated clusters using NCMine. Thirteen clusters containing YUC6 were generated; all clusters have been uploaded to NDEx (table S4).

**
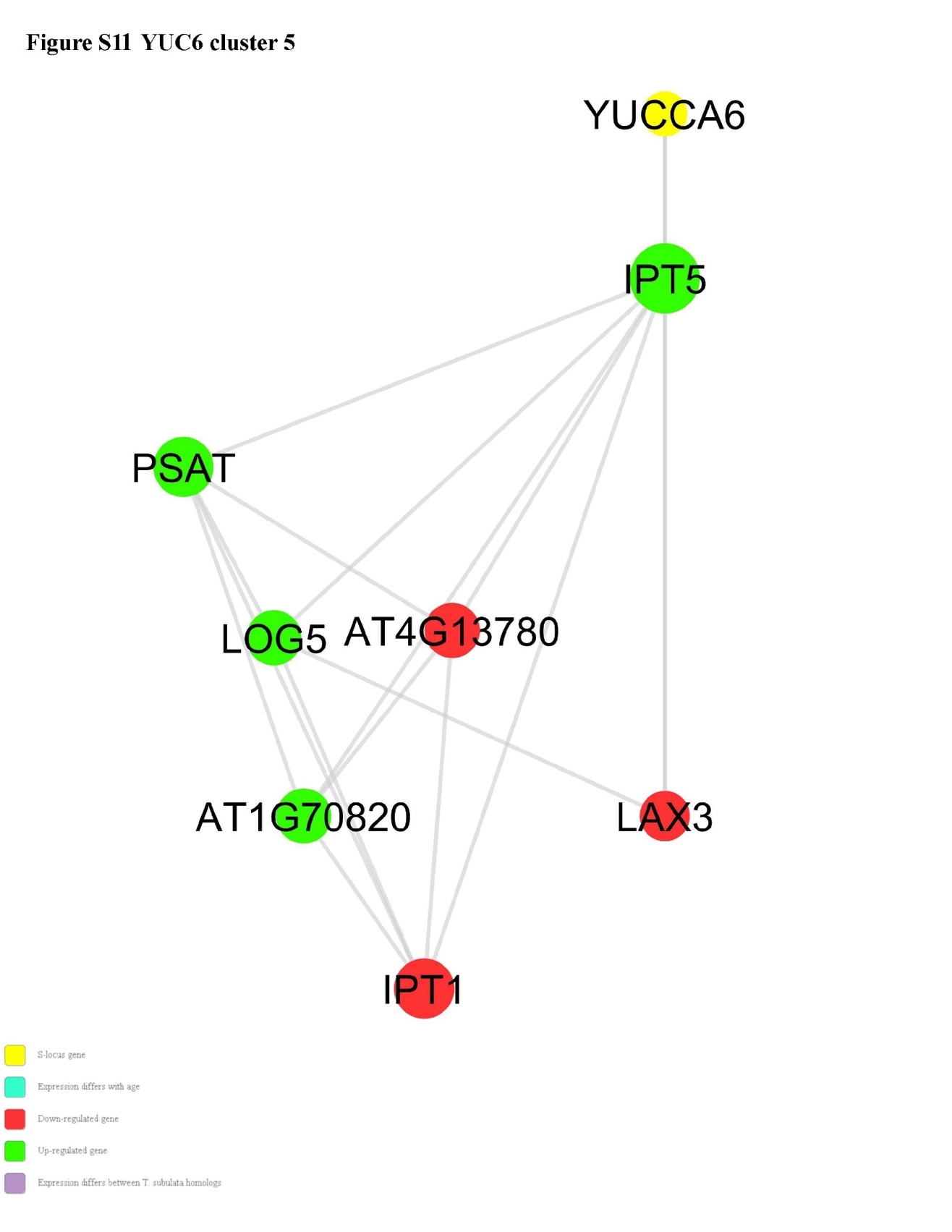
**

**Figure S11. YUC6 cluster 5.** To identify additional auxin related genes in our stamen DEG dataset, we generated clusters using NCMine. Thirteen clusters containing YUC6 were generated; all clusters have been uploaded to NDEx (table S4). Clusters 5 was of particular interest as it contains several auxin related genes and other phytohormone related genes.

**
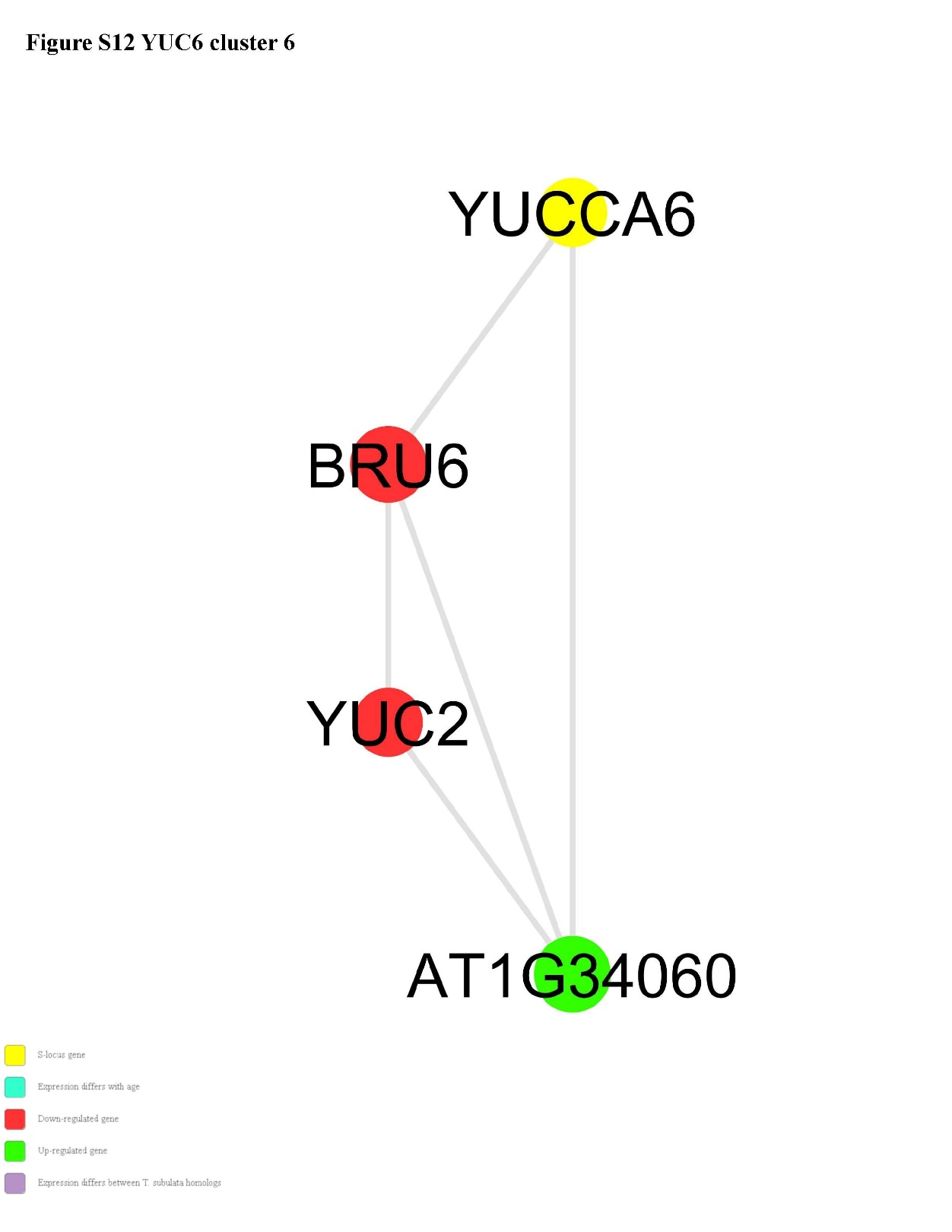
**

**Figure S12. YUC6 cluster 6.** To identify additional auxin related genes in our stamen DEG dataset, we generated clusters using NCMine. Thirteen clusters containing YUC6 were generated; all clusters have been uploaded to NDEx (table S4). Cluster 6 was of particular interest as it contains several auxin related genes and other phytohormone related genes. Cluster 6 suggests alternative auxin biosynthesis pathways are downregulated presumably as a result of negative resulting from auxin synthesis by TsYUC6.


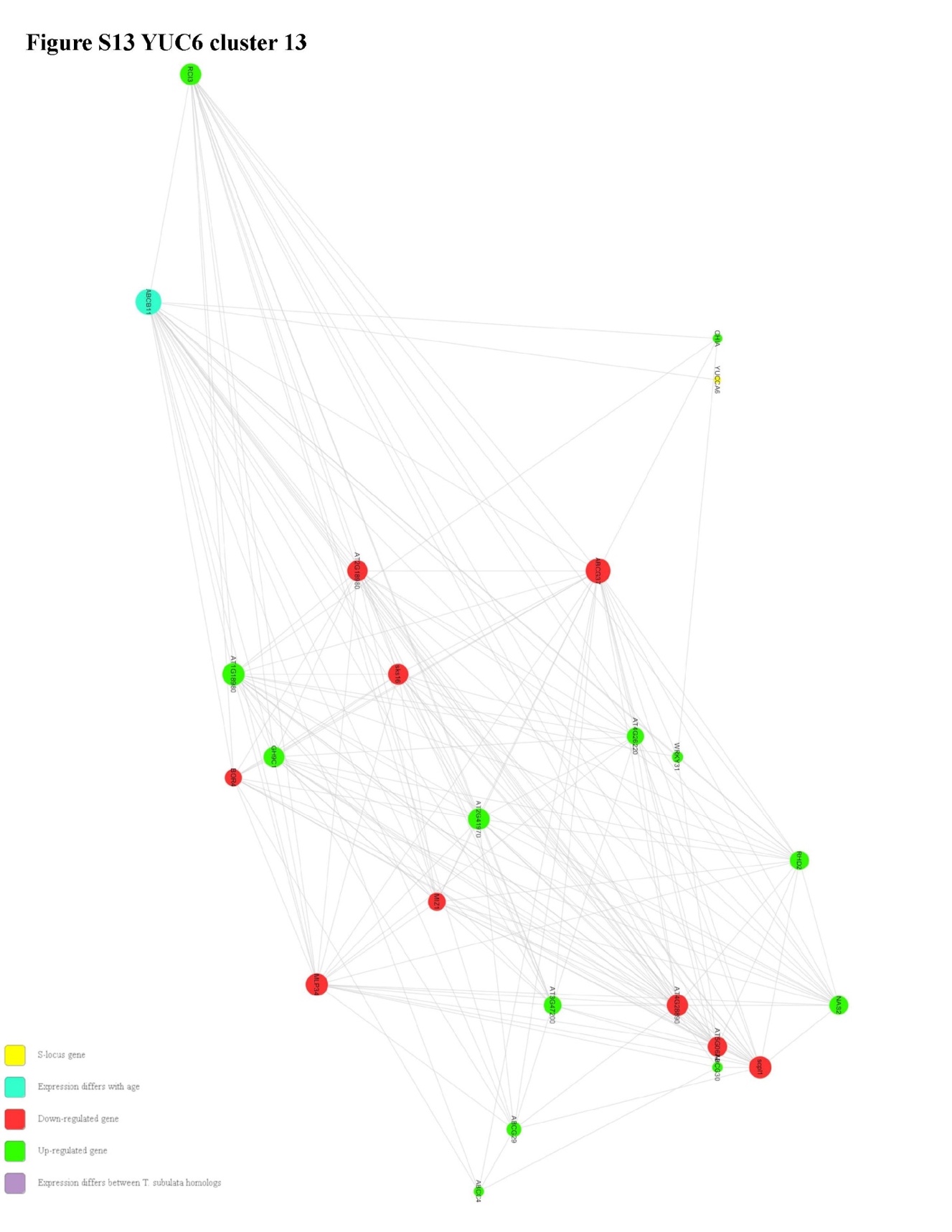


**Figure S13. YUC6 cluster 13.** To identify additional auxin related genes in our stamen DEG dataset, we generated clusters using NCMine. Thirteen clusters containing YUC6 were generated; all clusters have been uploaded to NDEx (table S4). Cluster 13 was of particular interest as it contains several auxin related genes and other phytohormone related genes.

**
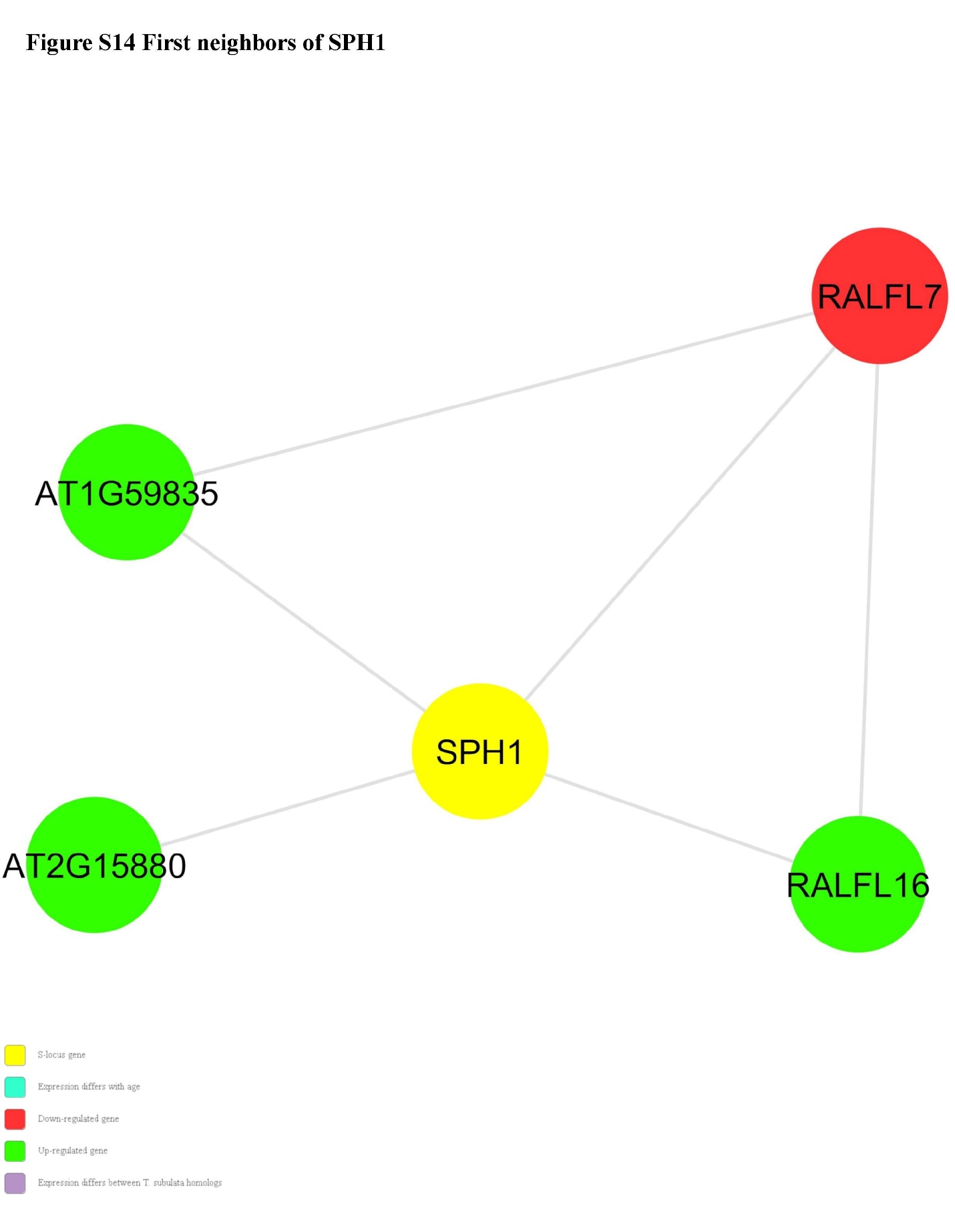
**

**Figure S14. First neighbors of SPH1.** First neighbors are genes that are predicted to associate directly with the gene of interest. STRING analysis identified four first neighbors of SPH1.

**
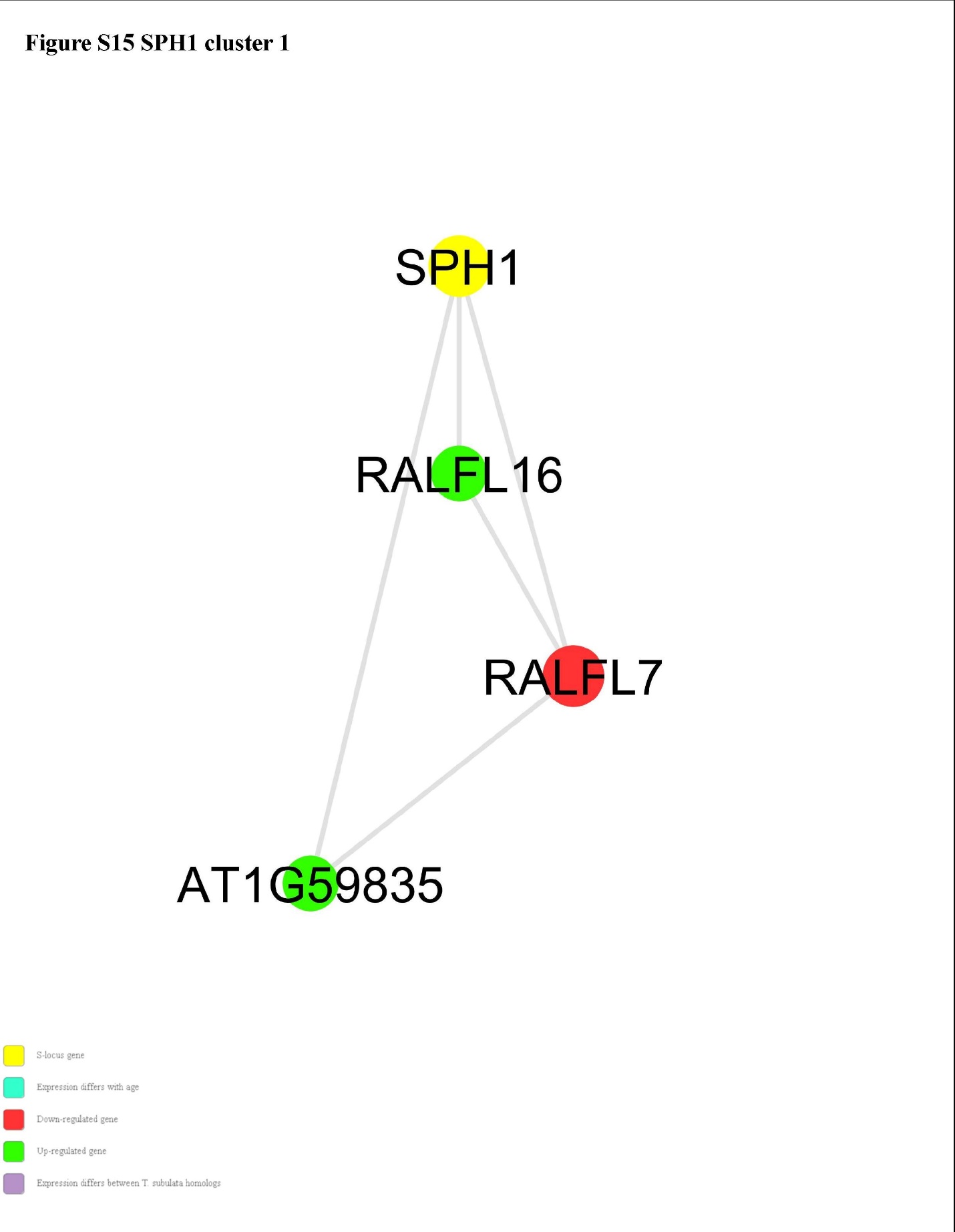
**

**Figure S15. SPH1 cluster 1.** As with the other S-locus genes, NCMine generated clusters containing SPH1 were identified. Only one cluster containing SPH1 was identified. Three of SPH1’s four neighbors made up this cluster. All were small signaling polypeptides.

1. Szklarczyk, D.; Gable, A.L.; Lyon, D.; Junge, A.; Wyder, S.; Huerta-Cepas, J.; Simonovic, M.; Doncheva, N.T.; Morris, J.H.; Bork, P.; et al. STRING v11, protein-protein association networks with increased coverage; supporting functional discovery in genome-wide experimental datasets. *Nucl. Acids Res.* **2019**, *47*, D607–D613.
2. Tadaka, S.; Kinoshita, K. NCMine Core-peripheral based functional module detection using near-clique mining. *Bioinformatics* **2016**, *32*, 3454–3460.
3. Pratt, D.; Chen, J.; Welker, D.; Rivas, R.; Pillich, R.; Rynkov, V.; Ono, K.; Miello, C.; Hicks, L.; Szalma, S.; et al. NDEx, the Network Data Exchange. *Cell Syst.* **2015**, *1*, 302–305.
4. Skuta, C.; Bartunek, P.; Svozil, D. InCHlib—Interactive cluster heatmap for web applications. *J. Cheminform.* **2014**, *6*, 44.
5. Aramaki, T.; Blanc-Mathieu, R.; Endo, H.; Ohkubo, K.; Kanehisa, M.; Goto, S.; Ogata, H. KofamKOALA, KEGG ortholog assignment based on profile HMM and adaptive score threshold. *bioRxiv* **2019,** doi:10.1101/602110.
